# Exploring the *raison d’etre* behind metric selection in network analysis: a systematic review

**DOI:** 10.1007/s41109-022-00476-w

**Published:** 2022-07-14

**Authors:** D. Morrison, M. Bedinger, L. Beevers, K. McClymont

**Affiliations:** School of Energy, Geosciences, Infrastructure and Society, Heriot-Watt University, William Arrol Building, Room W.A. 3.36/3.37, 2 Third Gait, Currie, Edinburgh, EH14 4AS UK

**Keywords:** Network analysis, Graph theory, Urban systems, Disaster management, Natural hazards

## Abstract

Network analysis is a useful tool to analyse the interactions and structure of graphs that represent the relationships among entities, such as sectors within an urban system. Connecting entities in this way is vital in understanding the complexity of the modern world, and how to navigate these complexities during an event. However, the field of network analysis has grown rapidly since the 1970s to produce a vast array of available metrics that describe different graph properties. This diversity allows network analysis to be applied across myriad research domains and contexts, however widespread applications have produced polysemic metrics. Challenges arise in identifying which method of network analysis to adopt, which metrics to choose, and how many are suitable. This paper undertakes a structured review of literature to provide clarity on *raison d’etre* behind metric selection and suggests a way forward for applied network analysis. It is essential that future studies explicitly report the rationale behind metric choice and describe how the mathematics relates to target concepts and themes. An exploratory metric analysis is an important step in identifying the most important metrics and understanding redundant ones. Finally, where applicable, one should select an optimal number of metrics that describe the network both locally and globally, so as to understand the interactions and structure as holistically as possible.

**Supplementary Information:**

The online version contains supplementary material available at 10.1007/s41109-022-00476-w.

## Introduction

As urbanisation increases, cities have become hubs of the modern world. Abundant with people, resources and services which drive the growth of technology, the economy and society; all of which are intertwined with one another (Cristiano et al. 2020). They operate as urban ‘systems’, and represent networks of different interacting and co-evolving constituent parts (van Meeteren 2019). It is estimated that 55% of the world’s population live in urban areas, with projected increases of up to 70% by 2050 (United Nations 2018). In order to maintain reliable functioning of the urban system, there will be associated increases in assets to accommodate this growing population. Expansion of the urban system in tandem with a changing climate leaves constituent parts (e.g. local economy; technical infrastructure) susceptible to potentially huge losses in the event of a perturbation (Bouwer et al. 2007; Alfieri et al. 2017).

Recent shocks to the system, have highlighted how complex and interconnected our urban systems really are. For example, the global outbreak of COVID-19 has impacted healthcare, education, employment, travel and wellbeing—all supported by individual systems that are reliant on each other. Understanding this ‘domino’ effect within urban systems is therefore paramount and risk management must progress by treating risks not in isolation, but instead by considering how risk permeates throughout the elements within the urban system as a whole. Past approaches to the management of urban systems have been critiqued as reductionist, i.e. where feedback effects and interactions between parts have not been sufficiently acknowledged (Cavallo and Ireland 2014). In such approaches, any risk posed to a constituent part was self-contained within itself, implying that it does not pose any risk to the wider system (Clark-Ginsberg et al. 2018). However, methods such as *Network Analysis* enable elements within a system to be mapped and linked together, presenting a more comprehensive approach to assessing urban systems.

**Fig. 1:**
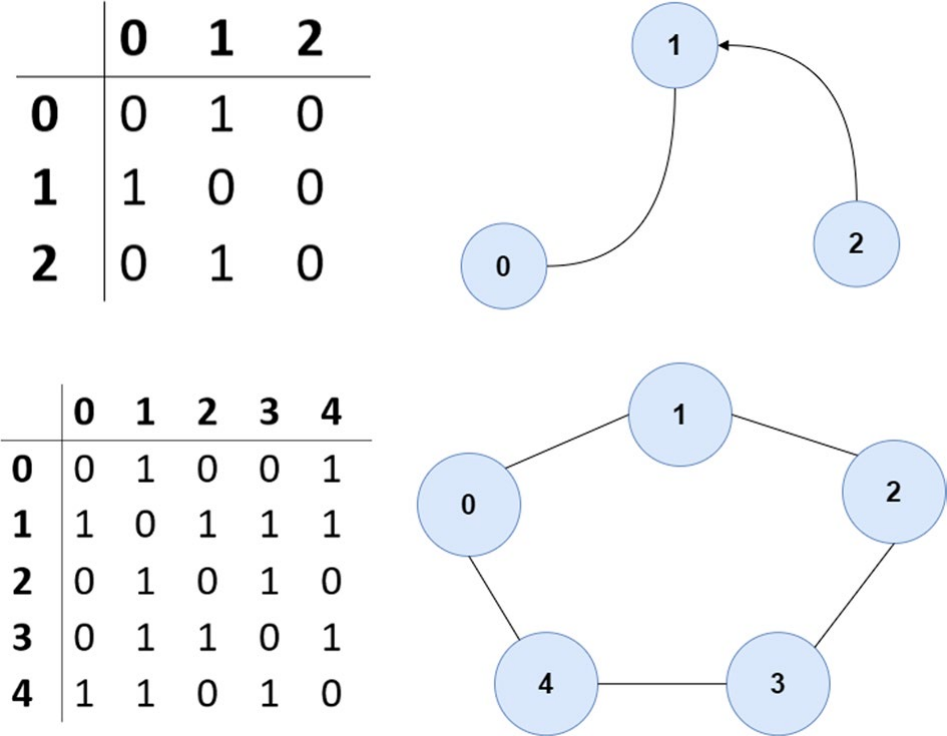
Adjacency matrix (left) to graph/network (right)

Network analysis is born from *Graph Theory*, where entities of a specified nature (e.g. services, people, houses, infrastructure, cities etc.) are represented as ‘vertices’ or ‘nodes’ that are each connected through a series of ‘edges’ or ‘links’. The use of graph theory for representing systems such as cities is relatively new, and is achieved through the following steps: (1) Identify network *typologies* (e.g. emergency facilities, households) (2) Define *connections* (e.g. emergency facilities provide relief to households and businesses) (3) Define *rules* (e.g. households and businesses are associated with their closest emergency facilities, such as fire stations, hospitals etc.) and (4) Build the *graph* in the form of an ‘adjacency matrix’ or ‘edge list’ (Fig. 1) (Arosio et al. 2020). When the graph is established, the structure and characteristics of the network can be analysed through an array of metrics.

Network metrics help to describe a network at either a local level (which looks at individual elements i.e. particular nodes or edges) or a global level (which looks at the network as a whole) (Miele et al. 2019). The most established and commonly used metrics are those developed by Freeman (1979): Betweenness Centrality, Degree Centrality and Closeness Centrality. The concept of centrality is one that pertains to the local level of a network, and seeks to quantify the position, importance or influence of an element in a network. Centrality originated in Social Network Analysis (SNA) (Newman 2010), therefore many characteristics that metrics such as Betweenness, Degree and Closeness describe are sociological in origin. However, centrality metrics have also been used to describe physical networks such as neural topology of the brain (Saberi et al. 2021); technical networks such as water distribution systems (Giustolisi et al. 2019); and multi-mode networks such as those modelling tangible and intangible elements in an urban system (Beevers et al. 2022; McClymont et al. 2021).

The transferability of centrality metrics across domains and contexts highlights a particular strength of network analysis in that many different systems, from the brain to entire cities, can be represented. However, Miele et al. (2019) argues there is a risk of blind use of metrics and other issues surrounding metric selection. The ease and availability of network analysis software allows end users to calculate a range of metrics without fully understanding the mathematics. It is therefore essential that the adopted metric is truly able to describe the characteristics of the system in question and is applied and interpreted by the researcher in an appropriate manner. Global metrics (e.g. Density, Diameter) are used to compare different networks, however simple characteristics that vary across each network (e.g. number of nodes) can influence the results of global metrics. In addition, multiple metrics are often chosen without clarification of how they each make different contributions to the analysis, which can lead to redundant results if the chosen metrics are in some way correlated.

Given the wide range of possible applications of network analysis, one might ask: when should I use a certain metric and not another? What are the reasons for choosing network metrics and is it fair to assume that common metrics (i.e. centrality) can consistently describe the same system characteristic across a wide range of scenarios and domains? How many metrics are appropriate to use? Are some metrics more versatile across contexts than others?

To explore these questions, this study looks at the wider urban systems and disaster management literature to find the *raison d’etre* behind metric selection in network analysis. We identify the most common types of network analysis and the supporting metrics to fulfil study objectives, therefore answering “*who uses what, where, and why?*”*.* This study aims to reflect on past selection and application of network metrics across the fields of urban systems and disaster management, to avoid misinterpretation of results due to inappropriate metric selection and maximise the usefulness of network analysis in future.

## Methodology

**Fig. 2:**
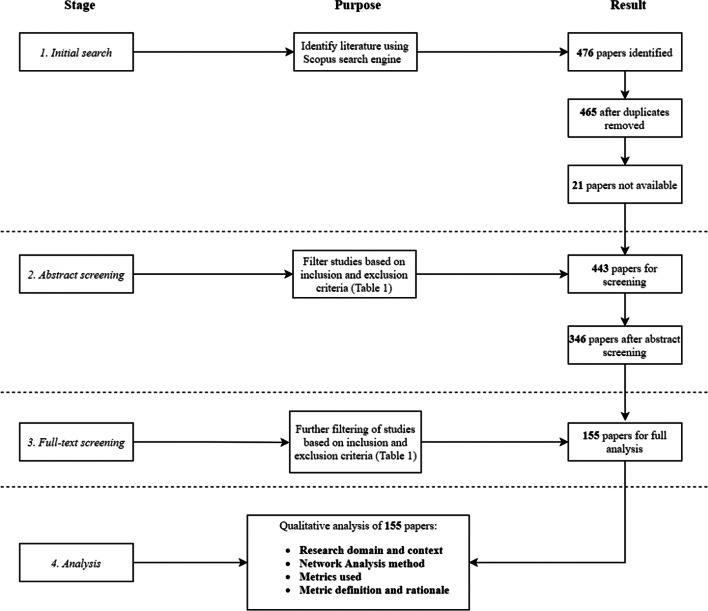
Research workflow

The materials used to conduct this review were retrieved from Scopus. Our analysis sought to identify research papers from the fields of disaster management and urban systems that feature network analysis. Papers were limited to journal or conference articles, written in English, with no upper or lower limit on publication date. In order to meet the PRISMA criteria of systematic reviews (Liberati et al. 2009) we outline the search terms used to systematically identify relevant literature and predefined inclusion and exclusion criteria to ensure reproducibility. The initial literature search was conducted on 16th March 2021 and the full research workflow is summarised in Fig. 2. Search queries in Scopus were as follows;

TITLE-ABS-KEY ("FLOOD MANAGEMENT" OR "DROUGHT MANAGEMENT" OR "DISASTER RISK REDUCTION" OR "DISASTER MANAGEMENT" OR "HAZARD MANAGEMENT" OR "URBAN SYSTEMS" OR "COMPLEX ADAPTIVE SYSTEMS") AND TITLE-ABS-KEY ("NETWORK ANALYSIS" OR "NETWORK THEORY" OR "NETWORK SCIENCE" OR "GRAPH THEORY" OR "GRAPH ANALYSIS" OR "GRAPH METRIC").

The initial search in Scopus returned a total of 476 papers, which after duplicates were removed was reduced to 465 papers. 21 papers were unavailable on Scopus for review therefore 443 papers were carried forward for Stage 2.

Strict inclusion and exclusion criteria were established to keep within the scope of this review (Table 1). These criteria act as a means in which to filter out studies as per Stage 2 of the research workflow, where the abstracts of the 443 papers were screened for eligibility. During the abstract-screening stage, papers were manually examined to determine whether they met the pre-defined eligibility criteria for the qualitative analysis. The primary purpose of this stage was to ensure that there was a technical application of network analysis that adopted one or more metrics. Papers that used network analysis as a form of visualisation only with no quantitative analysis were excluded. Moreover, Bayesian and neural network-based papers were excluded as these tend to be more events-based, focused on probabilities between events and extracting new insights from granular data through deep/machine learning. Here our focus is on structure-based network insights, e.g., whether one element of the network has more influence than another. The abstract screening process provided 346 papers for the full-text screening in Stage 3.

**Table 1:** Inclusion and exclusion criteria adopted to identify papers within the scope of the review

Inclusion criteria	Exclusion criteria
Studies that include a technical application of network analysis in the context of disaster management, urban systems and/or complex adaptive systems, to gain structure-based network insights	Studies that use “network” as a general descriptive term and do not explicitly include applications of network/graph theory Papers that adopt methods such as neural networks and Bayesian networks which aim to gain insights about probabilities of events Discourse papers on urban systems, complex adaptive systems, and disaster management

The full-text screening process in Stage 3 is an extension of the abstract screening stage. This stage involved full-text screening of 346 papers, in which the following information was documented in Excel; network method used, metrics adopted, research domain and context. Given that metrics that quantify centrality of a network have since been diversified, in that they extend out with SNA, it was necessary to distinguish between identified metrics and what particular branch of network analysis they were applied to. Moreover, distinguishing between research domain and context was also necessary since, for example, the disaster management domain comprises a wide range of contexts (i.e. different hazards and disasters). Out of 346 papers that qualified for full-text screening, 155 papers were identified for a richer, manual qualitative analysis. These papers served as the database that form the results and discussion of this review.

The final stage of the research workflow was to manually review the final 155 papers as comprehensively as possible using the documented information in excel as a guide, and also served to validate the initial information extracted during the full-text screening stage to minimise potential error. For example, each study had the adopted metrics recorded, therefore an aim of the analysis was to identify (if any) the rationale behind metric selection. Identifying the rationale behind metric selection for each of the 155 papers and comparing with the quantitative excel database (i.e. what metrics were used in what domain and context) facilitated answering the research questions. For instance, studies that share a research domain and context (e.g. examining the emergency response network after a flood) may adopt different metrics, or one study may adopt just one, whereas another adopts several metrics; is there an explicit reason for this? In addition, the characteristics that a metric is aiming to describe in a particular study were mapped to each metric for use in a frequency analysis. This aims to provide an understanding of how versatile metrics are in describing certain properties of a network. The literature search in this review covers multiple (albeit related) domains in its Scopus search (i.e. hazard management, disaster management, flood management, drought management + urban systems and Complex Adaptive Systems (CAS).

## Results

### Publication trend

**Fig. 3:**
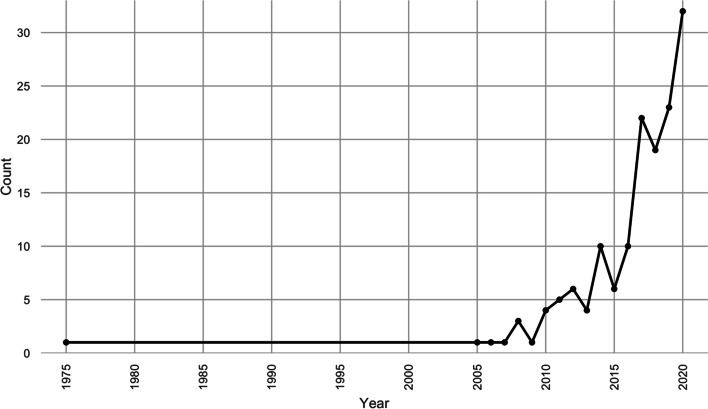
Network analysis publication trend

Figure 3 below outlines the timeline of network analysis applications in the 155 identified papers used in the analysis, as discussed in “Methodology” section. For this review, the earliest identified application of network analysis was in 1975. Following this, no applications of network analysis were featured until 2005. From then onwards, a sharp increase can be observed, with the largest annual peak in 2020 (32 applications). The sharp rise suggests that the value of network analysis is of increasing interest to researchers focused on extreme events. Conceptually, research in this area has increasingly acknowledged the systemic interconnectedness of events and impacts (Pederson et al. 2006). Practically, the influx of ‘Big Data’ from 2005 (van Rijmenam 2013) has created greater opportunities for insights into system behaviour, and facilitated analysis of more sophisticated networks, e.g. with geocoded social media data (Songchon et al. 2021).

Regarding the domains in which these studies are applied, 87 of the 155 (56%) papers analysed were within disaster management. The breadth of disaster management studies accounted for all hazards such as floods, tsunamis, earthquakes, hurricanes, terrorism, cyber-security and disease outbreaks. The remaining 44% of papers were related to “urban systems” and “CAS” and were diverse in their research domains, with applications in: sustainable development, supply-chain management, healthcare, transport and critical infrastructure, ecology and wider climate-related research such as water resources and emissions. The balance between network publications in urban systems and disaster management highlights an almost even application on short-term problems concerned with direct shocks, or disturbances (disaster management) and longer-term more complex problems such as urban planning in systems research.

### Network methods

**Fig. 4:**
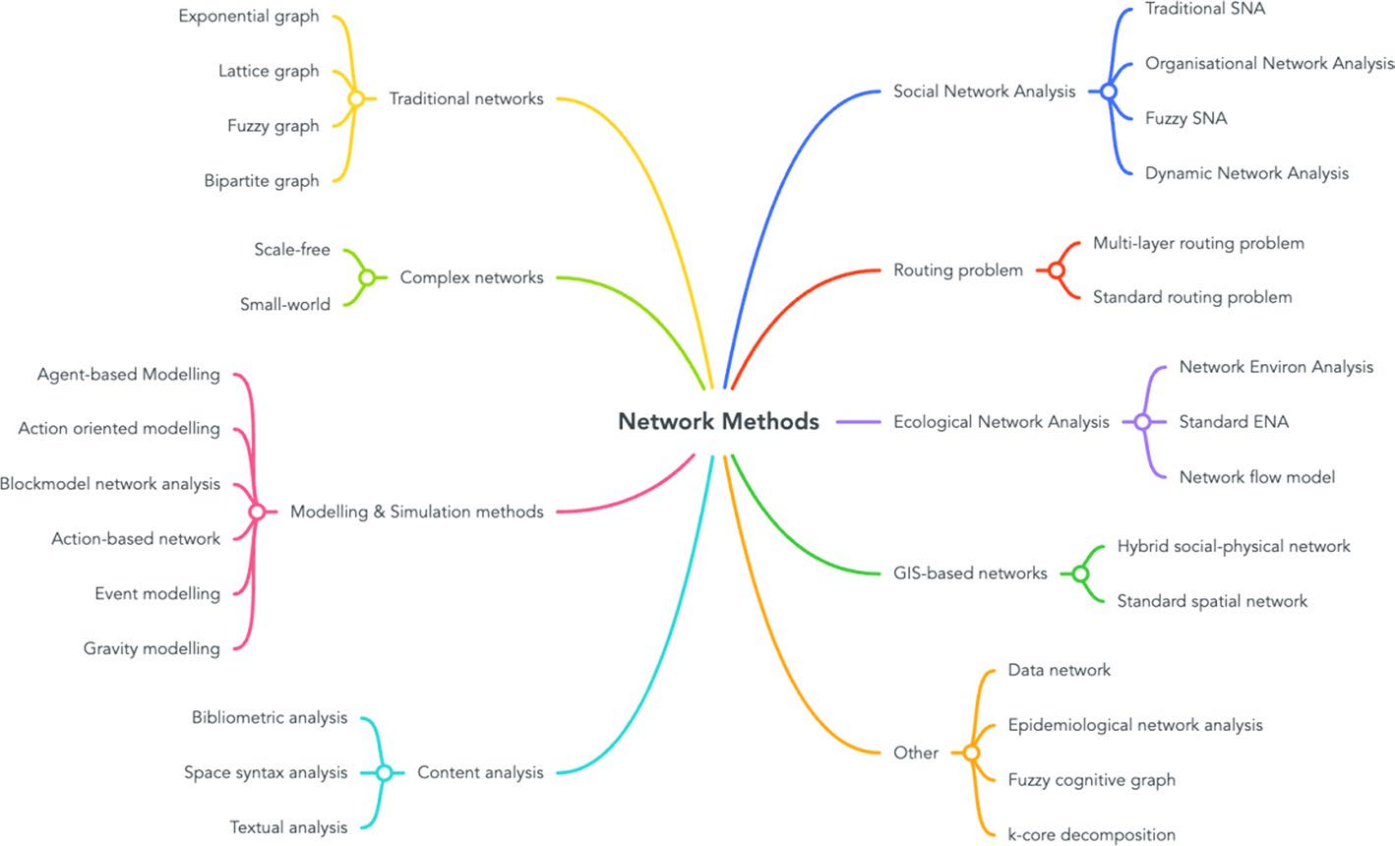
Map of identified network methods according to categories

In total, we identified 31 unique network analysis methods that can be grouped into 8 categories (Table 2). Figure 4 provides a breakdown of each individual method.

**Table 2:** Network analysis methods

Method category	Broad category definition	No. of papers	Percentage (%)^a^
Social network analysis	Networks that examine social structure of graph, where nodes typically represent actors/people	69	42
GIS-based network	A graph in which the nodes and edges are defined based on georeferenced data	22	13
Routing problem	Typically, the same as GIS-based networks, however main objective is finding optimal/shortest path in the network	20	1312
Ecological network analysis	Network methods that follow typical ENA approaches, where flows between systems/nodes are analysed	17	1210
Modelling/simulation	Computational model-based methods in which network analysis is incorporated	16	10
Standard network	Networks that follow standard structures	12	7
Complex network	Networks that follow complex structures	3	2
Content analysis	Textual/bibliographic based network methods	3	2
Other	Any method that does not fall into any of the above categories	4	2

^a^Some studies use multiple methods, therefore total % is > 100

The most popular method was SNA, accounting for just under half of all studies (42%, 69/155 papers). Applications of SNA were predominantly in the disaster management domain (65%, 45/69 papers), where the context was mainly focused on Emergency Management Networks (EMN) such as how Governments and Local Authorities responded to previous disasters. For more detailed information on networks in EMNs, we refer the reader to the review by Du et al. (2020).

The next most popular network methods are GIS-based networks (13%, 22/155 papers) and routing-problem-based networks (12%, 20/155 papers). Whilst we acknowledge that routing problem-based networks are also spatial in nature, we categorise them differently based on the method objective; GIS-based networks in this review refer to networks developed using georeferenced data that are diverse in research domain, context and aim. For example Ceron et al. (2020) examine meteorological topology of the system using radar-derive rainfall data and Sun et al. (2019) investigate regional economic development of urban agglomerations in China. On the other hand, routing-problem-based networks are exclusive within the disaster management domain (95%, 19/20 papers), where there is a focus on evacuation, emergency service response or access to relief facilities. One exception is a wider transport-related application of a routing problem by Guettiche and Kheddouci (2019) who examine critical transport nodes and links to mitigate congestion. Thus two of the most popular network analysis methods (SNA and Routing Problem methods) are almost exclusively focused on disaster management, it suggests that there is a lack of methodological diversity in the domain, with respect to network analysis techniques.

In contrast, 6 out of 8 method categories (approximately 51% of papers) are within the wider urban systems domains outlined previously. The modelling/simulation methods category consists of 6 different methods (Fig. 4) which incorporate network analysis with applications in critical infrastructure (Pumpuni-Lenss et al. 2017) and urban development (Sun et al. 2019). Moreover, simulation methods such as Agent-based Modelling (ABM) are integrated within SNA, routing problems and Ecological Network Analysis (ENA) (7 papers).

Applications of ENA are typically applied in the same area of urban and sustainable development (66%, 12/18 papers), with exceptions in ecology (Borrett et al. 2006), climate (Chen et al. 2015) and aviation (Burns et al. 2008; DeLaurentis and Ayyalasomayajula 2009).

Celik and Corbacioglu (2018), Comfort et al. (2013) and Jin et al. (2014) feature as examples of typical ‘complex networks’, mainly small-world and scale-free networks. Both applications of complex network featured in this review are exclusive to disaster management.

### Network metrics

In total 38 global metrics and 41 local metrics were identified from the qualitative analysis of 155 papers, with a pool of 79 metrics available overall. Additional file 1: Table S1 provides a summary table with the definitions of each metric and a classification as either “Global” or “Local”. Global metrics typically describe characteristics, topology, and structure at the network/system level. Conversely, local metrics are typically centrality-based measures that describe the characteristics of a network at the node/edge level. The split between global and local metrics is roughly even, where out of all 79 metrics, global metrics account for 48% and local metrics 52%.

#### Local metrics

Overall, the top three most frequently occurring local metrics were Betweenness Centrality (51%, 79/155 papers), Degree Centrality (32%, 50/155 papers) and Characteristic Path Length (i.e. mean path length or shortest/optimal path; 28%, 44/155 papers). Developed by Freeman (1979) (alongside Closeness Centrality; the 4th most frequently occurring metric. 15%, 23/155 papers) the concept of centrality was developed in the context of social networks. Given that SNA is also the most popular form of network analysis (every application of SNA utilises at least one centrality metric), it is no surprise that they are the most popular metrics. Similarly, the prevalence of GIS-based networks and Routing problems (53%, 42/79 papers) highlighted in “Network metrics” section can also explain the popularity of path length as a metric, as the applications of routing problems are typically associated with evacuation behaviour, and thus shortest and optimal path lengths are the objective in these studies (see Xuefen and Lim 2016).

**Fig. 5:**
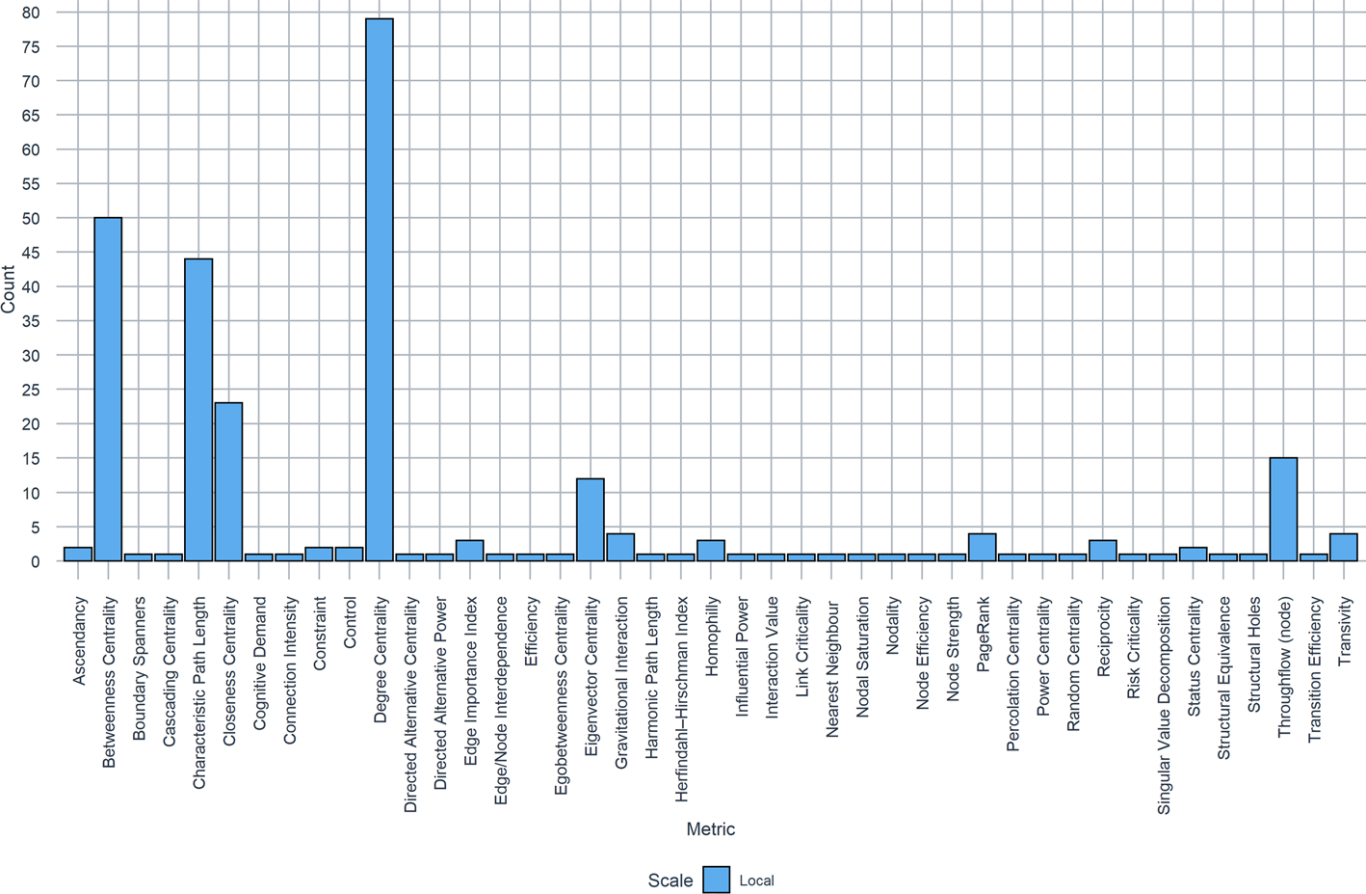
Frequency of local network metrics

Whilst centrality metrics are most popular where SNA is concerned, of particular interest is how these metrics were applied across the other categories of network analysis described in Table 2, and in what domains. Of the papers that did not use SNA, 40% (34/86 papers) adopted centrality measures, highlighting that these metrics are versatile out with social networks. Beyond the established centrality which dominate local analysis, there are 8 additional centrality metrics that cumulatively account for < 1% (7 papers). These are as follows: Cascading Centrality and Random Centrality (Der Sarkissian et al. 2020), Directed Alternative Centrality and Directed Alternative Power (Zheng et al. 2020), Egobetweenness Centrality (Hossain and Kuti 2010), Percolation Centrality (Dong et al. 2020), Power Centrality (Radulescu et al. 2020), and Status Centrality (Ongkowijoyo and Doloi 2017; Tang and Lai 2019). All but three of these centrality measures (Directed Alternative Centrality, Directed Alternative Power and Power Centrality) were applied in the context of disaster management using either spatial networks or SNA, whereas the other three were applied in urban systems in the context of smart cities (SNA) or examining spatial interaction, migration and inter-city flows (spatial network). Although all these studies have deviated from the norm by including lesser-known centrality metrics, only Zheng et al. (2020) did not supplement their analysis with one of either Betweenness, Closeness or Degree Centrality. Figure 5 summarises the distribution of local metrics.

#### Global metrics

**Fig. 6:**
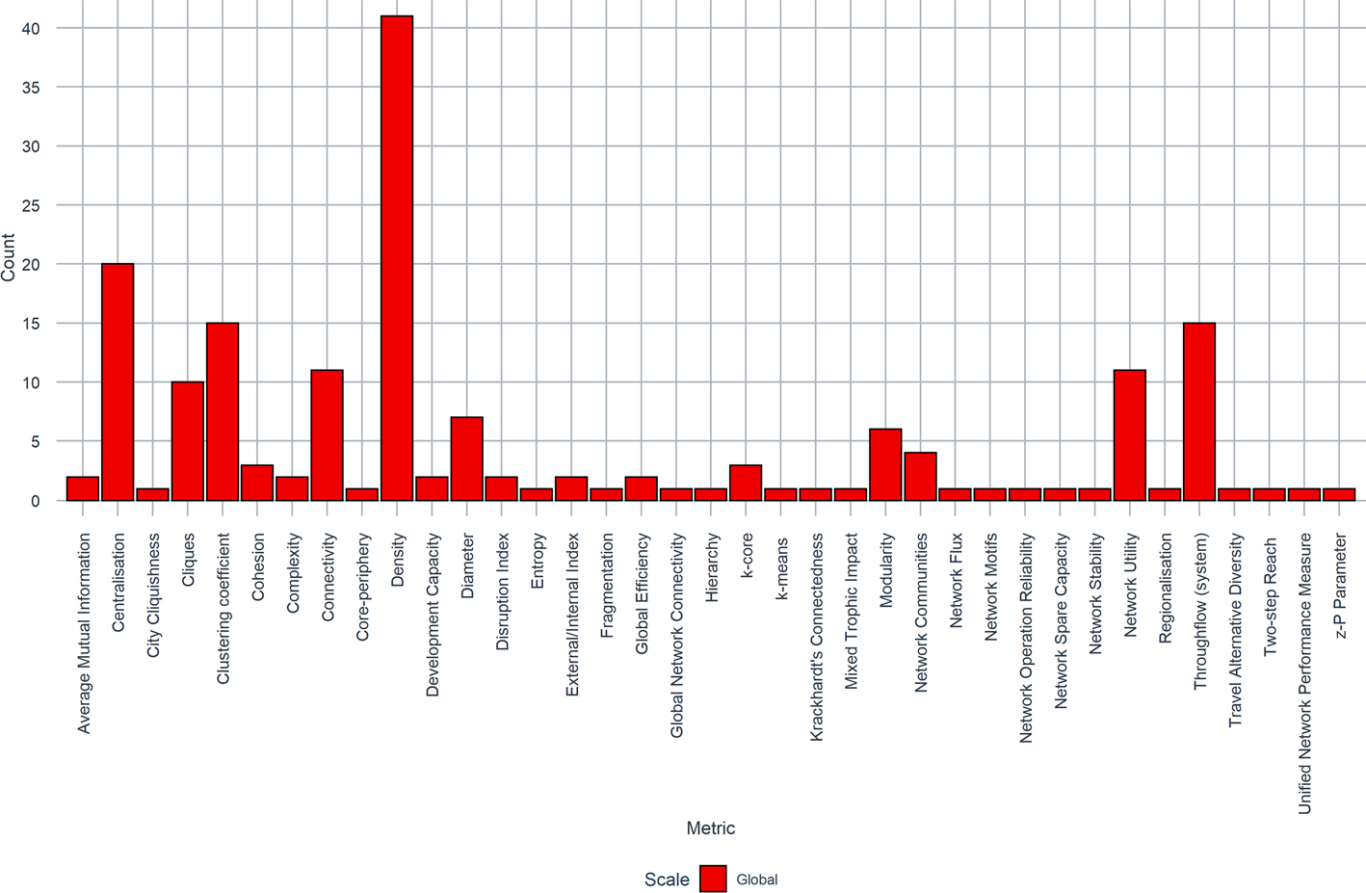
Frequency of global network metrics

Figure 6 illustrates the most popular global metrics. The top three most popular global metrics were Density (26%, 41/155 papers), Centralisation (13%, 20/155 papers), and Throughflow (9.7%, 15/155 papers) joint with Clustering Coefficient (9.7%, 15/155 papers). Density and Centralisation are complementary ‘global' metrics to Betweenness and Degree Centrality, as they provide an overview of a network as a whole, as opposed to the centrality of individual nodes. Largely these metrics are applied in SNA. There are few instances in which Density (7 papers) and Centralisation (2 papers) were adopted out with SNA. For example, Wang et al. (2020) adopt density as a topological measure of a Human-Spatial system based on geocoded social media data before, during and after Hurricane Harvey. He et al. (2019) use Density to assess the hierarchical structure of China’s ‘megaregions’ to explore the urbanisation process.

In addition to established metrics such as Degree, Betweenness, Density and Centralisation, there are several bespoke applications of metrics that are unique to particular studies. In an exploratory analysis of the ‘World City Network’, Derudder and Taylor (2005) present ‘Global Network Connectivity’ (GNC) which is an aggregated measure of a city’s connectivity in relation to other cities and ‘City Cliquishness’, which is derived from an already popular means of assessing the structure of a network through ‘clique analysis’ (6%, 10/155 papers).

Similar to the points raised in “Local metrics” section, where centrality metrics are largely associated with applications of SNA, the same can be said for ENA. This approach is built around network ‘flows’ (Throughflow), in which the flows (e.g. energy, resources, passengers) are used to analyse the interaction between multiple systems (Fath et al. 2007). Throughflow is both a global (system level) and local (node level) metric (Borrett et al. 2006; Finn 1980). Whilst ENA is always associated with flows there are instances where ENA has been supplemented with additional metrics which are also unique to ENA: Average Mutual Information (AMI), Development Capacity and Ascendency (Bodini 2012; Bodini et al. 2012), Network Utility (e.g. Gao et al. 2021), Control (Tan et al. 2018; Yang et al. 2020), Network Flux (Liu et al. 2011), Stability (Fan and Fang 2019) and Mixed Trophic Impact (Gao et al. 2021).

#### Number of metrics used

**Fig. 7:**
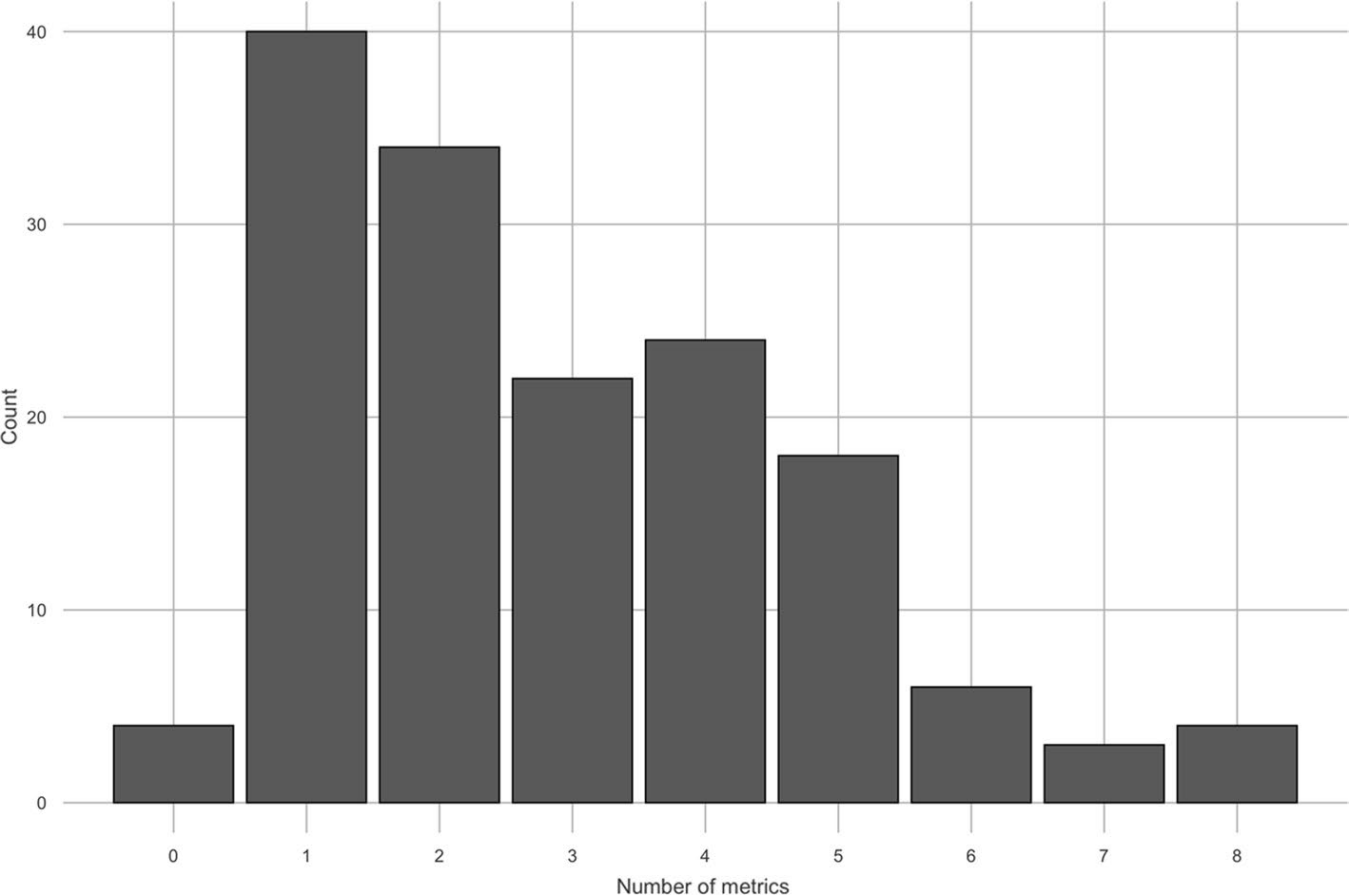
Distribution of metrics based on how many metrics are adopted across the 155 papers.

Figure 7 below summarises the distribution of metrics across the 155 papers. The distribution is right-skewed, indicating that a majority of papers adopted 1–2 metrics in a network analysis, with an average metric count of approximately 3.

At the extremes of the distribution, there are 4 occurrences in which there were no explicitly stated metrics adopted in the analysis. For example, Pastor-Escuredo et al. (2020) adopt SNA in the context of Flood Risk Management (FRM) to propose a rapid multi-dimensional impact assessment framework using social media data. In contrast, another SNA-based approach by Ongkowijoyo and Doloi (2017) adopts 8 metrics: Density, Centralisation, Degree Centrality, Closeness Centrality, Betweenness Centrality, Eigenvector Centrality, Status Centrality and Risk Criticality. The emphasis towards local level metrics suggests that various characteristics of the nodes within the network are necessary to describe. Romascanu et al. (2020) highlight that historically, consistent centrality measures are not adopted when identifying central nodes within networks. When examining this in the context of this review, it is found that 17% of SNA studies adopt only one metric. This therefore begs the question as to why some applications of network analysis do not aspire to describe the system in question as holistically as possible, through a suite of metrics which describe different aspects of the network.

Whilst these examples are based on SNA approaches, it is important to highlight that the number of metrics adopted is context dependent, with respect to the chosen network method. For example, ENA is unique in that it does not require the same indicators as SNA as it focuses on flows, as opposed to characteristics such as centrality. Therefore the typical metrics adopted in this approach are Throughflow (at both system and node level) or Network Utility (with additional metrics adopted as highlight previously in “Global metrics” section). Similarly, Routing Problems are less concerned with characterising the important nodes, instead the shortest/optimal route is the priority, therefore Characteristic Path Length is often the only metric adopted (70%, 14/20 papers).

### Network characteristics

The previous section, “Network methods” and Network metrics” aimed to give an overview of the state-of-the-art regarding network methods and metrics. This section explores the extent to which the rationale behind use of particular metrics is reported and made transparent. In other words: what do we hope to learn about systems through network analysis? Why are particular metrics used, and what key concepts or system properties are thought to be targeted by each?

In a small proportion (6%, 10/155 papers) of papers there was a lack of transparency and explicit reporting around the rationale behind metric selection. This suggests that there is no particular ‘blind use’ of metrics with the vast majority of papers providing a description of how the metric relates to the system in question, or at least a generic definition. Of the papers where no explicit rationale or definition is provided however, the typical metrics adopted are those which featured most frequently in “Local metrics” section (Degree Centrality, Betweenness Centrality) and “Global metrics” section (Density, Centralisation, Clustering Coefficient). This suggests that their level of establishment is commonplace, such that it is assumed they require no detailed explanation.

**Fig. 8:**
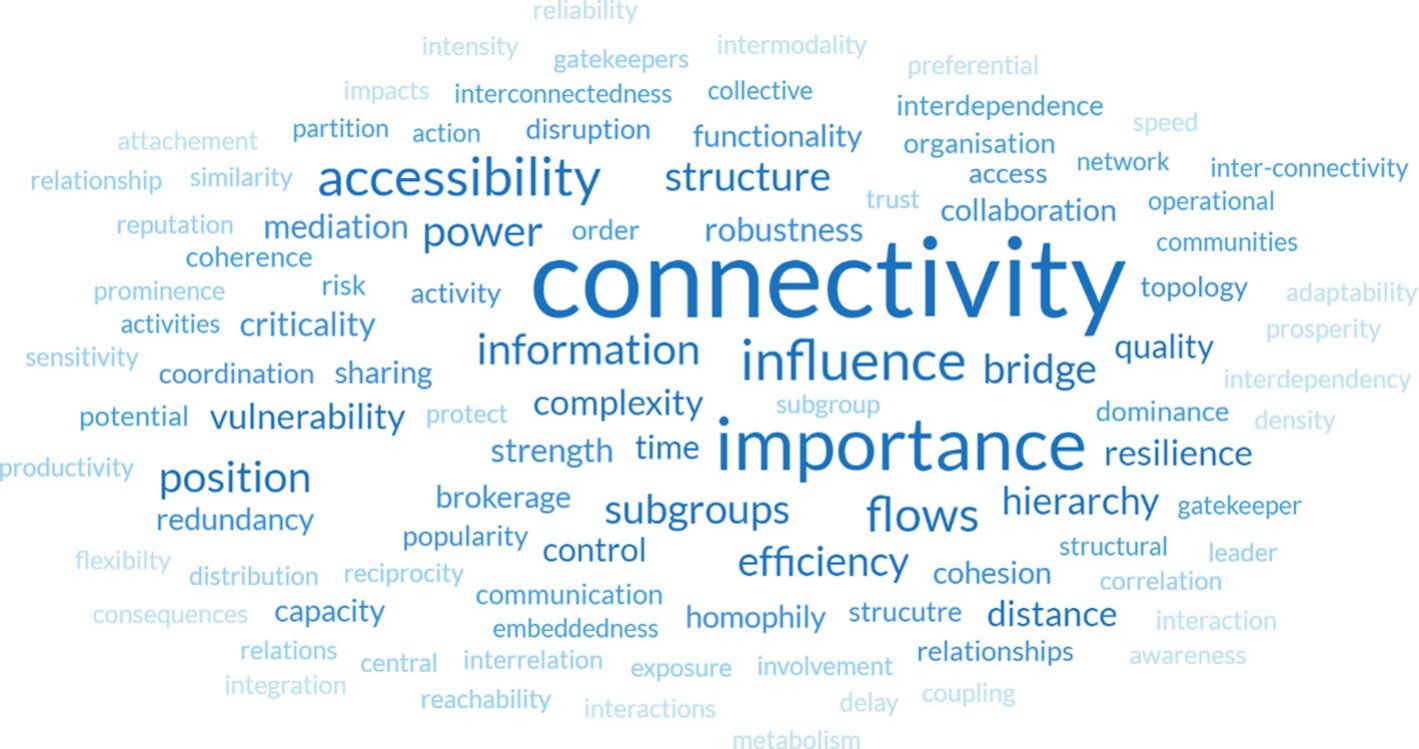
Word cloud revealing the most common characteristics described by metrics

Figure 8 illustrates the most frequently occurring characteristics described by the identified metrics. Table 3 provides a summary of the top 8 characteristics with their frequencies. It is no surprise that the most frequently occurring characteristic is *connectivity*. In total, 12 metrics were described as measures of connectivity. In some cases, the distinction between metrics of connectivity are related to the scale of the network being measured. For example, Degree Centrality can measure node (local) connectivity, whilst Cohesion is a measure of system (global) connectivity. It is clear therefore that it is less of case that connectivity is a priority in network analysis, but more the *nature* of the connectivity that is important (Fig. 8).

**Table 3:** Top 8 characteristics with frequency

Characteristic	Frequency	Associated metrics
Connectivity	34	Characteristic Path Length, Betweenness, Degree, Closeness, k-core, Clustering Coefficient, Disruption Index, Cohesion, Global efficiency, Centralisation, Global Network Connectivity, Density
Importance	20	Betweenness, Degree, Closeness, Eigenvector, Edge Importance Index, Link Criticality, PageRank, Node Interdependence
Accessibility	12	Characteristic Path Length
Influence	12	Betweenness, Degree, Closeness, Eigenvector, Status
Flows	10	Betweenness, Degree, Closeness, Edge Interdependence, Node Strength, Throughflow
Information	8	Betweenness, Degree, Closeness, Edge Interdependence, Node Strength
Position	8	Betweenness, Degree
Power	8	Betweenness, Degree, Closeness

The interchangeability of characteristics that metrics can describe is also highlighted in Table 3. Betweenness, Degree, Closeness and Eigenvector Centrality are all mentioned as describing a node’s *importance*, *influence*, *position* and *power*. Given that all of these metrics are mathematically different, it suggests that there are different ways in which a node can be “important” or “influential”. Furthermore, the main centrality metrics feature in most of the characteristics described such as being measures to describe “information flows”, therefore network metrics are highly diverse and context dependent.

## Discussion

### Who uses what? And where?

With respect to “who” is using network analysis amongst the targeted areas in a broad sense, the applications of network analysis reviewed here were somewhat evenly spread across disaster management (with respect to all kinds of hazards) and urban systems CAS literature (a breadth of contexts). When disaggregating this further by specific network analysis techniques, it is clear that there is a wide range of different methods available. This highlights that in the areas of disaster management and urban systems, network analysis has been significantly diversified since the original conception of SNA in the 1970s (see Zhang 2010).

Advances in computational power and data availability have not only facilitated more advanced applications of SNA, but have allowed integration with other computational methods such as modelling and simulation. For example, Rodrigueza and Estuar (2018) use SNA as a basis for understanding disaster behaviour in an ABM. Furthermore, network analysis has evolved beyond the study of sociology, as the importance of transport and critical infrastructure has become a modern concern. For example, georeferenced data has enabled path analysis of transport and mobility, or the “actors” (nodes) in a network no longer need to be people or organisations, but instead businesses, homes and emergency facilities in “Hybrid-social physical networks” (Bozza et al. 2017). Furthermore, graph theory has facilitated systems-oriented methods such as ENA, which not only pertains to ecosystems, but the interactions between physical systems such as cities across the world (Bodini et al. 2012).

With respect to “who uses what”, despite the availability of a wide range of specific network analysis methods, there is siloing within the reviewed research domains. A majority (65%) of disaster management studies used either SNA or Routing Problems. However, the application of network methods is broader in the urban systems domain, where 6 out of 8 method categories (Table 2) were used in 51% of studies overall. The focus on SNA and routing-problem-based networks within disaster management suggests that there are established priorities, yet also highlights gaps where network analysis may also be able to fill. For instance, the primary application of SNA is centred around response networks and organisational collaboration during previous disasters, where two extreme events (i.e. floods) are compared to examine how networks have evolved between two past points in time. This is intended to examine the preparedness of a nation or region. However, the prevalence of such applications suggest that there may be an overemphasis on understanding past events, instead of more directly preparing for future events. Applications that are more present- or future-oriented (i.e. evacuation and emergency service response modelling across spatial transport networks) do suggest that there is some element of future preparation involved, however, as they are based on shortest/optimal path problems, they mostly represent preparedness in the context of spatial movement, rather than abstract social collaboration. Because looking to future preparedness requires dealing with a great deal of uncertainty regarding how context may change from the present, and it is crucial to understand the more fundamental (but highly complex) dynamics behind preparedness in the present before introducing those future uncertainties, these findings are understandable. However, the high prevalence of SNA or Routing Problems suggests more could be done to expand the conceptualisation of research problems—and diversify the application of network analysis techniques—within disaster management (Bedinger et al. 2019). This review suggests that disaster management should therefore turn towards the wider urban systems literature for inspiration regarding alternative network analysis methods that consider the interdependency of multiple systems as opposed to mobility only. For instance, ENA applications in urban systems studies often model the interactions between different sectors (Liu et al. 2011).

With respect to “where”, we have further disaggregated this review by the focus of specific network analysis techniques—in other words, whether the metrics used have a local or global perspective. A wide range of metrics were identified in the chosen research domains with an almost equal representation between global and local.

It should be acknowledged that this discussion emphasises centrality metrics, due to their overwhelming popularity, and that a comprehensive discussion of reviewed applications for each of the 79 metrics would be cumbersome and out with the scope of this study.

Unsurprisingly, the most frequently used of all metrics (both global and local) were all local metrics of centrality (Freeman 1979): Betweenness, Degree, and Closeness. These are sociological in origin, and have coevolved with the development of SNA, whereby they have typically been used to describe social entities. However, the diversification of network analysis has led to a diversification of metrics in two ways. First, SNA is no longer only “social”—it is the go-to network analysis method regardless of the phenomenon being studied. Although centrality metrics are grounded in SNA, and thus would be assumed to pertain to sociological entities, these metrics have been used to describe a host of other entities, or to describe economic proxies whilst the main method is still SNA. This is important as it begs the question as to why in disaster management there remains such a focus on applying SNA to mainly social-based networks, rather than extending this to different sectors that are interconnected and are at risk (e.g. health care, economy, transport). Second, centrality metrics are frequently applied in methods other than SNA (e.g. ABM), and the concept of centrality has developed beyond metrics proposed by Freeman (1979) to other centrality metrics.

The versatility of centrality metrics and the availability of many other metrics (global and local) highlights the value of network analysis. Although this does present issues as well.

### How many metrics should I use? When should I use this metric and not the other?

It is challenging to definitively answer how many metrics one should use in network analysis, as this depends on context, adopted method, time, resources, and knowledge of the end user. However, based on the results in “Number of metrics used” section, three things are clear; the average number of metrics observed in this review is three; the majority of studies adopt fewer than 3; and there is wide variation across the studies (i.e. some studies adopt several (8) and some adopt none at all). Centrality metrics are typically used to capture specific characteristics of a network, such as evaluating how a single node is connected to the rest (degree centrality), which provides a static overview of network structure. From a more dynamic perspective, betweenness centrality evaluates how ‘information’ propagates through the network. Other centrality metrics, such as eigenvector centrality aim to fill the gaps of basic nodal metrics such as degree centrality, as it includes ‘information’ (such as a nodes influences) whilst also describing the connectivity as degree centrality evaluates. Given these three perspectives, this could possibly explain why typically studies returned in this review adopt an average of three metrics. This would therefore assume that there is a minimum number of characteristics required to evaluated a network.

If the purpose of having an array of metrics is to capture different characteristics of a network, then it could be argued that more metrics are better, as each metric would contribute to holistically describing the system. However, this is where context becomes important. For example, Cui and Li (2020) aimed to measure two concepts: social capital and how it is used in community resilience. Both of these concepts are multi-faceted and represent complex sociological interactions, such as sense of belonging, collective efficacy, trust, and reciprocity. To achieve this, Cui and Li (2020) adopt one global metric (Density) and seven local metrics (Betweenness, Degree, Closeness, Path Length, Efficiency, Constraint, Structural Holes) as appropriate to these concepts. In a different context, Balsiger and Ingold (2016) aimed to investigate how actors within flood governance collaborate and share information based on perceptions of sustainability, using just one local metric (Degree Centrality). Degree Centrality uses the concept of “Structural embeddedness” (see Granovetter 1992) which describes how embedded an actor is in the network based on how central they are (i.e. how many actors they are connected to). Both studies clearly define their objectives and achieve them through appropriate metrics; the former study’s scope is wider or more complex, therefore a wider range of metrics is perhaps necessary.

However, one could also use the latter study to highlight inconsistent metric applications between studies measuring similar concepts. Balsiger and Ingold (2016) and another study (Comfort et al. 2016) both aim to examine collaboration. Comfort et al. (2016) use another sociological concept: “bridging” actors. These are defined as actors that link between two indirectly connected actors, and this can be measured using Betweenness Centrality. If the objectives of the two studies are similar, why has one adopted Betweenness Centrality and the other has not? Further contradicting these observations is Faas et al. (2017), whose objective is also analysing bridging actors, however in this instance, Degree Centrality is the only metric presented in the paper. These examples show it is difficult to justify which and how many metrics should be used, based only on referencing past applications of disaster management and urban systems research, because the existing body of work is inconsistent.

So how should we justify which and how many metrics should be used? The fundamental aims of network analysis are arguably to represent complex concepts (e.g. multi-faceted social interactions) with a systems perspective (i.e. what is happening both locally and globally). Selecting only one metric is insufficient to achieve either. One metric can only cover one concept, and either global or local characteristics. Therefore at least one global and one local metric is desirable. In addition, more is not necessarily better, as this runs the risk of redundancy if the results of the chosen metrics are correlated (Miele et al. 2019).

Therefore, we would argue that an important step in selecting network metrics is a correlation analysis in order to minimise this risk. For example, the R package, *Central Informative Nodes in Network Analysis* (CINNA) (see Ashtiani et al. 2019) enables comparisons across numerous measures of centrality to identify the most important metrics using Principal Component Analysis (PCA) and pairwise associations.

### Are some metrics more versatile than others? Can common metrics consistently describe the same characteristic across contexts?

The results in Sects. “Network metrics” and “Network characteristics” sections highlight that there is diversity in terms of what characteristics of a system or entities can describe. Moreover, the above discussion has alluded to versatility amongst metrics in that two metrics can describe the same thing and that terminology is interchangeable. This raises the question, does interchangeable terminology represent versatility? Or inconsistency in reporting? Furthermore, beyond the most common metrics, what about the less popular ones?

In favour of the argument of inconsistency is the fact that there were studies (albeit a small percentage) which provided no rationale or explanation of metric choice. Katerndahl (2012) uses SNA to understand how research collaboration within academic faculties impacts productivity at the individual and departmental level, however does not provide any definition or rationale behind the use of Degree, Betweenness and Eigenvector Centrality. Similarly, Comfort et al. (2013) and Oh (2017) provide no rationale for their selection of metrics. Kim and Hastak (2018) provide no rationale for Density, yet describe Degree, Betweenness and Eigenvector centralities as metrics to explain “prominence or importance”, without distinguishing how these three centrality metrics differ and why all three are required to measure the same concept. In contrast, Liu and Lim (2016) provide no definitions for the centrality metrics Betweenness and Degree, yet provide definitions and interpretations of Centralisation and Density. Moreover, Comfort and Zhang (2020) explain the rationale behind Betweenness Centrality and the External/Internal Index, but omit any explanation of Density or Diameter. Tozer and Klenk (2019) use only Degree in a Bibliometric analysis but provide no rationale as to what it represents. Ma et al. (2020) simply state that degree measures structure. Finally, Pheungpha et al. (2019) and Zelenkauskaite et al. (2012) do not specify which metrics or measure of centrality is being used, respectively. In these instances, it appears the analysis was qualitative and that the relationships (i.e. who was connected to who) was of primary interest. Rather than specific failures to adequately outline methodological choices, we believe these instances speak to a larger issue of “letting the researcher decide” how to communicate about network analysis in non-mathematical fields. This is a barrier to a more transparent, higher standard of interdisciplinary network science.

In terms of versatility, it appears that it is not necessarily always a case of *which* characteristic is being measured but a case of *how*. The most frequently occurring characteristic is *connectivity.* In the context of Routing Problem based methods, this typically refers to how connections between nodes are disrupted as a result of a hazard in which the optimal path length is impacted due to a loss of connectivity (Espada et al. 2015). *Accessibility* is also a frequently appearing characteristic which is interchangeable with connectivity in this context. Connectivity is used as a generic term when adopting centrality metrics, in which Degree, Betweenness and Closeness describe different aspects of connectivity. For example, Čerba et al. (2017) describe the connectivity of semantic resources in terms of quantity (Degree), distance and relation (Closeness) and whether nodes act as *bridges* (independent, or indirectly connected nodes; described by Betweenness) or not. Optimal connectivity is therefore described as a node which is connected to many others, acts as a bridge and is close to each other nodes. However, whilst in this instance connectivity appears to be a characteristic described by three measures of centrality, there are several examples of this that do not relate to well-known and oft-used centrality metrics. Derudder and Taylor (2005) use the GNC metric to measure a city’s connectivity in relation to other cities. This metric does not consider centrality. Furthermore, “Connectivity” is also a metric that represents the minimum amount of nodes or edges that would need to be removed to fragment the network into two or more isolated subgroups (Diestel 2005) and Samarasinghe and Strickert (2013) claim that Density is a global indicator of connectivity. It is no surprise that connectivity is the most frequently occurring characteristic, given that networks are fundamentally about connection.

Similarly, the same applies for *importance* and *influence.* Kim and Hastak (2018) state that Degree, Betweenness and Eigenvector centrality are used to explain the importance of actors in an SNA analysis of social media data post-disaster. Taking a selection of examples from the application of SNA to measure response networks in disaster management, Calliari et al. (2019) use Degree to assess the *influence* of the most central actors in the network, Celik and Corbacioglu (2018) use Degree to highlight the most *important* and well-connected actors, and Celik and Corbacioglu (2016) and Cui and Li (2020) both measure the *power* of actors using Degree. Moreover, Celik and Corbacioglu (2016) use Betweenness as a means of measuring an actors’ *position* in the network, yet Htein et al. (2018) measure such actor-level *positioning* using Degree (with respect to Centralisation). Mathematically, Degree Centrality is simply the number of other nodes which a given node is connected to (Freeman 1979). Therefore, it is recognisable in the context of social networks that a well-connected actor plays a prominent role in the network, and possesses influence and importance. However, the nature of this influence and importance is not only a function of the amount of connections. For instance, Meilani and Hardjosoekarto (2020) and Chen et al. (2020) make a distinction between Degree and Eigenvector Centrality by stating that power is measured in the latter not by how *many* connections a node has, but *who* the connections are. In both examples, nodes represent actors within disaster risk reduction efforts after an event and the *power* is identified by examining nodes who are both mutually high in Eigenvector Centrality, thus identifying *who* is most powerful differently than Degree Centrality affords.

Whilst centrality metrics are most popular where SNA is concerned, of particular interest is how else these metrics have been applied. A number of studies adopted centrality measures out with SNA, and described entities other than people or organisations. For example, Lao et al. (2016) use degree centrality to weight edges in their network to represent air passengers, thus providing a measure of a city’s centrality. Arora and Ventresca (2018) use Betweenness and Closeness centrality for preferential linking in the synthesis of resilient Supply Chain Networks (SCN), where centrality measures act as proxies for price, performance and quality. Mu et al. (2020) examine the spatial distribution of green space and physical factors to explore alternative green space planning strategies using Degree, Closeness and Betweenness. Garrett et al. (2017) adopt Degree, Closeness, Betweenness, Eigenvector as measures of centrality to explore food security and agricultural networks (alongside Cliques, Diameter and Path Length). In disaster management, centrality measures are typically associated with road networks and critical infrastructure; Fan and Mostafavi (2019) use degree centrality with social media data in a graph-based event detection model to identify disruption of critical infrastructure. Papilloud et al. (2020) characterise flood exposure of road network using Edge Betweenness Centrality (EBC) and Sasabe et al. (2020) also apply EBC in road network risk analysis. Alongside Lao et al. (2016), these instances were the only three in which Betweenness Centrality was measured at edges instead of nodes.

Whilst interchangeable terminology for some metrics is a prevalent theme emerging from this review, there are instances in which the metric being described is more definitive. A metric that is terminologically consistent across studies is Throughflow, used in ENA. Whilst the nature of the flows may vary between study, the purpose of applying the metric remains the same. Throughflow is classed as both a global and local metric. Locally, the flows can measure the importance of a node, whereas at a system level, the Total System Throughflow (TST) can indicate if a system is at a steady state if the sum of all inflows is equal to outflows. Measuring TST indicates the level of activity that pertains to the system in question, and this can be useful to characterise the system’s level of growth (e.g. economic growth in a city) (Bodini et al. 2012). This presents a useful insight into the methods of network analysis as it is clear that SNA has evolved beyond sociology in terms of method and metrics, and its background in sociology has perhaps fostered the level of versatility, interchangeability, and at times, ambiguity as to what metrics actually mean for those interpreting their results. ENA on the other hand is far more clear-cut, as it depends on measuring flows in terms of materials and resources, not the roles of individuals which are far more difficult to quantify.

Moreover, there is also more consistency and less ambiguity in studies that adopt bespoke/composite/less popular/less generalisable metrics. Nakatani et al. (2018) demonstrate adaptability of network analysis by using a well-established economic indicator, the Herfindahl–Hirschman Index (see Matsumoto et al. 2012) to measure the vulnerability of supply-chains. In contrast to vulnerability (an assessment of weak network links) is criticality, which measures importance (Knoop et al. 2012). Mitsakis et al. (2016) adopt the Unified Network Performance Measure (UNPM) to assess the performance of a transportation network against technological and natural disasters. Developing on the approach by Nagurney and Qiang (2008), the UNPM is an example of a metric that has been developed to measure the performance of a network in a specific context, therefore the meaning of “importance” in comparison to that described by centrality measures is less ambiguous. An additional example of bespoke metrics are Travel Alternative Diversity and Network Spare Capacity Dimension by Xu et al. (2018). These are measures of redundancy in a transport network and aim quantify alternative travel routes and how much spare capacity the network has under normal and disruptive conditions.

## Conclusions

### Summary of findings

The modern world is highly complex and comprises of numerous interconnected entities. Understanding the interactions between these parts is of great importance if the fragility of the urban system is to be understood and mitigated. To do so requires advanced analytical methods that are able to quantify the importance of a particular component and examine its role in the system with respect to other parts upon which it may rely. Network methods have significantly increased in popularity as the availability of data and specialised software enables such analyses. Network analysis is not a restrictive technique as it can be applied across various contexts and domains, such as understanding the key actors and processes in social networks to supply-chain management. It is highly diverse, even beyond the scope of this review which covers disaster management and urban systems exclusively. Within the confines of these research domains, this review highlights some key issues in network analysis related to: the range of network methods in disaster management and urban systems, the selection of appropriate metrics to describe characteristics and whether or not metrics versatile, and how many metrics are necessary for a holistic analysis.

First, the concepts of graph theory and network analysis have developed well beyond original applications (SNA). It has developed into a research domain of its own and has become highly diverse in terms of what problems it seeks to answer; spanning from analysis of social systems, ecological systems, economic systems and infrastructure to name a few. In addition, it has purpose as a standalone method, but also as a supplementary method to simulation and modelling problems, such as human behaviour. Furthermore, SNA remains the most popular method, however extends beyond social entities. However, despite the diversity, siloing exists between the research domains examined in this review. Answering “*who uses what, and where?*”, we find that the majority of this diversity is found in the wider urban systems literature. Disaster management could take inspiration from this domain as it may benefit from applying SNA more broadly, out with the contexts of EMN. Furthermore, methods such as ENA may be appropriate in the context of systems-oriented approaches. Network analysis coupled with ABM is gaining traction. It should also be emphasised that whilst we discuss methodological diversity, this is constrained to the two specified research domains examined as part of this review. As a result, for network analysis applications more broadly, there are likely be additional methods that this review has not covered.

Second, answering “*who uses what, and why?*”, we find that the centrality metrics have evolved in tandem with the methodologies. Sociological in origin, centrality metrics are not constrained to quantifying the properties and characteristics of social entities. They can be applied to networks describing cities and air passenger flows. However, the versatility of these metrics has led to diverging meanings and interchangeable terminology. This makes it difficult to underpin an appropriate metric to analyse networks with, as it may be the case that the same metric describes one characteristic in one instance, and then something different in the other. Furthermore, out with centrality, there is a vast range of metrics that have been developed for a specific purpose, making them ‘bespoke’. Metrics can also that utilise established indices coupled with network principles (such as centrality) to form composite metrics. This review identified 79 metrics in total, many of which are not discussed, however it is clear that there are many metrics that are not being used.

Finally, answering the *raison d’etre* further, knowing when to select one metric and not the other, alongside how many metrics to adopt in an analysis is a point of importance. This review finds that the average number of metrics adopted is three, however nearly 20% of papers adopt only one. Given that metrics can either be categorised as global or local, selecting only one metric potentially fails to capture the necessary characteristics of the network. In contrast, there are instances where studies adopt up to eight metrics in an attempt to capture *all* characteristics. However, this approach falls short due to the fuzzy lexicon of certain metrics in that multiple are reported to describe the same thing. This potentially results in redundant analysis and multicollinearity between metrics.

### A way forward

In light of the above findings, we outline a way forward researchers embarking on network analysis. The steps outlined acts as points to consider when approaching problems that require analysis of a network.

1. *Define target concepts and/or frameworks that support the study objectives.* It is then possible to map these concepts to the entities in the network and establish what characteristics are being measured. Characteristics can then be mapped to appropriate metrics. We refer the reader to supplementary material for a full list of the metrics identified in this review and definitions. One can also cast the net wider and use the CINNA package in R (see Ashtiani et al. 2019) which provides metrics beyond those identified in this review.
2. *Make a metrics shortlist.* Ensuring your target concepts are in line with a shortlist of possible metrics by reviewing applications of similar metrics in the research domain of interest, make a shortlist of metrics for testing.
3. *Perform an exploratory metric analysis.* Using packages like CINNA, it is possible to perform PCA and Correlation analysis on a multitude of variables. PCA describes which of the shortlisted metrics contribute to the analysis most and describe how much of the variance within results. Correlation analysis is a quick way of identifying redundant metrics. Revise shortlist if necessary.
4. *Adopt at least more than one metric and understand the maths.* Mapping concepts such as social characteristics to mathematical formulas is difficult and it is possible that one may interpret the results different from what the maths describes. It is therefore important that in the final analysis, more than one metric is adopted to get an idea of how interpretation of results may change depending on the metric outputs in question. Furthermore, where possible, describe the network at both local and global scales.
5. *Be explicit.* Outline explicitly the rationale behind metric selection, what they aim to measure and describe, how the maths translates to the target concepts and rules of interpretation.

## Supplementary Information

**Additional file 1. Table S1.** Network metric definitions.

## Data Availability

Not applicable.

## Abbreviations

ABM: Agent-based modelling
FRM: Flood risk management
CAS: Complex adaptive systems
CINNA: Central informative nodes in network analysis
ENA: Ecological network analysis
EBC: Edge betweenness centrality
EMN: Emergency management networks
GNC: Global network connectivity
PCA: Principal component analysis
SNA: Social network analysis
TST: Total system throughflow
UNPM: Unified network performance measure
