# Supplementary material for: Exploring the raison d’etre behind metric selection in network analysis: a systematic review
Source: Appl Netw Sci. 2022 Jul 14;7(1):50. doi: 10.1007/s41109-022-00476-w (PMC9281375; doi:10.1007/s41109-022-00476-w)
Supplement: Supplementary file 1 — Additional file 1. Table S1. Network metric definitions. [file 41109_2022_476_MOESM1_ESM.docx]

# **Additional file 1**

## S1 – Network metric definitions

| **Metric** | **Scale** | **Definition** |
| --- | --- | --- |
| Ascendancy | Local | Ascendancy is the product of two other metrics used in Ecological Network Analysis; Average Mutual Information (AMI) and Total System Throughflow (TST) (Ulanowicz, 1986; Tribus and McIrvine, 1971). It measures the of medium (e.g. flows, such as water or energy) that a system distributes in an efficient way. It is indicative of system growth and development (Bodini, 2012; Bodini et al. 2012) |
| Average Mutual Information | Global | Average Mutual Information (AMI) estimates how orderly and coherent flows are connected in a system; systems with high AMI values are said to be highly organised (Bodini, 2012; Bodini et al. 2012). It is indicative of system maturity (Ulanowicz, 1980; Odum, 1969). |
| Betweenness Centrality | Local | Betweenness Centrality is a measure of how often a node lies on the shortest path between other nodes (Freeman, 1977) |
| Boundary Spanners | Local | Boundary Spanners are nodes that the link distinct clusters or groups of nodes. These nodes act as a “bridge” for information flow and is measured using Betweenness Centrality (Hanson & Ford, 2010) |
| Cascading Centrality | Local | Cascading Centrality is the calculated centrality as a result of intentional, target removal of nodes in descending order of their weight and degree, starting with the highest connected nodes first (Ghanbari et al. 2018). Degradation of the network in this way assesses the resilience and vulnerability of a network in terms of connectivity (Der Sarkissian et al. 2020). |
| Centralisation | Global | The Centralisation of a network is indicative of how central its most central node is, relative to how central the other nodes in the network are (Freeman, 1979). |
| Characteristic Path Length | Local | Collective term that accounts for analysis of the length of edges; average path length, shortest path, optimal path (e.g. quickest travel time). |
| City Cliquishness | Global | City Cliquishness is a relative measure of a nodes cliquishness, based on the number of cliques it is a member of and its Global Network Connectivity (Derudder & Taylor, 2005).  See "Cliques” and “Global Network Connectivity”. |
| Cliques | Global | A Clique is defined as a maximal set of actors in which every actor is connected to every other actor (Derudder, 2005). In social network analysis, a clique is thus defined as a set of nodes in which each is connected to each other (Scott, 1991). Also referred to as “Subgroups”, “Subgraphs”, “Clusters” or “Communities”. |
| Closeness Centrality | Local | Closeness centrality indicates how close a node is to all other nodes in the network. It is calculated as the average of the shortest path length from the node to every other node in the network (Golbeck, 2013). |
| Clustering Coefficient | Global | The Clustering Coefficient describes of how well connected a network is in terms of how nodes cluster together, developed by Watts and Strogatz (1988) to indicate whether or not a graph is a “Small-world network”. |
| Cognitive Demand | Local | Cognitive Demand identifies nodes in a network that are well connected with a large number of links to other nodes (see “Degree Centrality”), however lack a level of connectivity to knowledge and resources to fulfil their objectives (Carley et al. 2011; Reminga and Carley, 2003). Unique to Social Network Analysis software, ORA. |
| Cohesion | Global | Cohesion refers to the level to which there are distinguishable subgroups or cliques within a network (Calliari et al. 2019). Cohesion effectively gives a characterization of how uniform or fragmented a network is (Cavalcanti and Giannitsarou. 2017). Fully connected networks show a cohesion index of 1 and disconnected networks are below 1.  See also "Cliques" and “Network Communities”. |
| Complexity | Global | Complexity is a composite measure and is an indicator for integration and cohesion. It is equal to the ratio of links present in a network versus the total number of possible links (March 2001; Sorenson, 2003). |
| Connection Intensity | Local | In contrast to Degree Centrality, it can be used to explicitly describe the spatial evolution of intercity connections. Connection intensity is the total number of linkages between two nodes (He et al. 2019). |
| Connectivity | Global | Connectivity is one of the basic concepts of graph theory. It is measured based on the minimum number of elements that would need to be removed before the network is fragmented into two or more isolated subgraphs (Diestel, 2005). Connectivity is an important measure of a networks resilience.  See also “Cliques”, “Cohesion”, and “Network Communities”. |
| Constraint | Local | Constraint characterises the 'closedness' of a network, that is, Closeness of a node in the network directly or indirectly with other nodes (P. Cui & Li, 2020). A lower value of Constraint is indicative of structural holes.  See also “Structural Holes”. |
| Control Analysis | Local | Control Analysis is indicative of the level of control and dependency between components or systems in Ecological Network Analysis (Schramski et al. 2006). |
| Core-periphery | Global | Core-periphery reveals the position and role of each node in an urban polycentric network. The value of "Coreness" determines whether the urban nodes in the systems should be classified as core areas or marginal zones (Wang et al. 2020). Indicative of network structure. |
| Degree Centrality | Local | Degree centrality is the simplest centrality measure to compute. A nodes degree is simply a count of how many connections (i.e. edges/links) it has. The degree centrality for a node is simply its degree. A node with 10 connections would have a degree centrality of 10. A node with 1 edge would have a degree centrality of 1 (Golbeck, 2015). Density is sometimes referred to as the *local* Clustering Coefficient (Golbeck, 2013).  See also “Density”. |
| Density | Global | Network density refers to a measure of the prevalence of dyadic linkage or direct ties within a network, where a 'dyad' refers to a pair of actors (nodes) and is the smallest structure of a social network (Frey, 2018). |
| Development Capacity | Global | In Ecological Network Analysis, Development Capacity describes the maximum potential that a system has to achieve further development and serves as an upper boundary for system organisation (Bodini, 2012; Bodini et al. 2012). |
| Diameter | Global | Diameter is the longest distance between any two nodes in a network (Makagon et al. 2012) |
| Directed Alternative Centrality | Local | Directed Alternative Centrality (DAC) is calculated based on the difference between in-degree (total links coming in to a node) and the out-degree (total links going out). In the context of World City Networks, if the DAC is positive it is indicative of an agglomerative city, otherwise it is diffusive (Zheng et al. 2020; Neal, 2011)  See also “Directed Alternative Power”. |
| Directed Alternative Power | Local | In comparison to DAC, the DAP reflects cities’ ability to control or affect the fluxes of resources and is used to identify four types of cities: 1) quintessential cities, both central and powerful, 2) hub cities, central but not powerful, 3) gateway cities, powerful but not central, and 4) peripheral cities, neither central nor powerful (Zheng et al. 2020; Neal, 2011). |
| Disruption Index | Global | The Disruption Index is a network level measure that indicates the degree to which connectivity is impact between origin-destination node pairs in the context of transport (Murray-tuite, 2007; Murray-Tuite and Masmassani, 2005). |
| Edge Importance Index | Global | The Edge Importance Index (EII) is an indicator of a population that can traverse along a particular link/edge (Chakraborty et al. 2018). |
| Edge/Node Interdependence | Local | Edge Interdependence is indicative of intermodality (Hu et al. 2020; Strano et al. 2015). Node Interdependence quantifies the additional value of interlayer coupling to the importance of cities in an urban system network (Hu et al. 2020). |
| Efficiency | Local | Efficiency is a measure of the degree of influence a node has over other nodes in the network. It refers to the ratio of the size of the point to the size of the point relative to the whole network (P. Cui & Li, 2020). |
| Ego-betweenness Centrality | Local | In the context of ego-networks, Ego betweenness of a particular node is the betweenness of that node in relation to its immediate neighbours (Everett & Borgatti, 2005). |
| Eigenvector Centrality | Local | Eigenvector centrality is a measure of the influence of a node in network, while giving consideration to the importance of its neighbours. For example, a node with 300 relatively unpopular friends on Facebook would have lower eigenvector centrality than someone with 300 very popular friends (like Barack Obama) (Golbeck, 2013). |
| Entropy | Global | Entropy is used to measure the structural and topological complexity of networks (Shannon, 1948). Regular networks have low entropy, whereas random networks are highly complex, and thus have high entropy. Network entropy can also be considered as a quantitative measure of network robustness (Zuo & Kajikawa, 2017). |
| External/Internal Index | Global | The External/Internal Index is the ratio between external and internal interactions in a network and measures the extent to which a network’s performance can be characterised by in-degree and out-degree ties (Krackhardt and Stern, 1988). |
| Fragmentation | Global | Fragmentation measures the numbers of nodes in a network that are not connected to other nodes in comparison to the total number of dyadic nodes in the network; the higher the value, the more fragmented a network is (Comfort et al. 2020; Chen et al. 2007)  See also "Cohesion". |
| Global Efficiency | Global | Global efficiency of a spatial network is defined as the average of the normalised pairwise efficiency over all possible pairs of nodes (Vragovic and Diaz-Guilera, 2005). |
| Global Network Connectivity | Global | Global Network Connectivity is a high-level measurement of the connectivity of cities in relation to other cities in the context of World City Networks (Derudder & Taylor, 2005). |
| Gravitational Interaction | Local | Gravitational Interaction is an output from Gravity models. The gravity model is based on Newton's law of gravity, in which the attractions between two nodes is proportional to their mass and inversely proportional to their respective distance (Stewart, 1948). Gravity models are a well know method for studying spatial interactions, such as economic trade between two cities (Von Gnechten et al. 2020).  See also "Interaction Power". |
| Harmonic Path Length | Local | Harmonic mean path length (HMPL) accounts for disconnected dyads (Newman, 2003). Harmonic mean distance between a vertex and all others, also called "efficiency" of the vertex (Latora & Marchiori, 2003). |
| Herfindahl–Hirschman Index | Local | The Herfindal index is an economic concept that is used to determine a company’s position in terms of market concentration (Laine, 1995) |
| Hierarchy | Global | The network hierarchy measures the degree to which a subset of nodes propagates risks to others in the network. The higher value of network hierarchy indicates that the hierarchical structure characteristic of the network is of a greater degree, and the directivity of risk propagation progress is more evident (Tang & Lai, 2019). |
| Homophily | Local | Homophily in Social Networks is a term used to describe nodes that interact with each other consistently, due to similarities - "similarity breeds connection" (McPherson et al. 2001). Homophily can be measured using the assortativity coefficient (Newman, 2002) coefficient (Newman 2002). |
| Influential Factor | Local | Influential Factor (IF) is a composite indicator. It is developed to substitute population as “mass” in a Gravity Model. The IF encompasses a large number of socioeconomic variable. It is a single index that represents a distillation of these variables to best describe a city’s competitiveness and influential power (Han et al. 2018). |
| Interaction Value | Local | The interaction value considers the bi-directional nature of flows, as well as the weighting of the flows by the origin and destination masses, making it a quasi-gravitational measure (Burns et al. 2008)  See also “Gravitational Interaction”. |
| *k*-core | Global | The k-core network measure can be used to describe network characteristics that Degree distribution is unable. IT helps to reveal structural and hierarchical properties of the network (Seidman, 1983; Gao et al. 2020) |
| *k*-means | Global | *k-*means refers to *k*-means clustering. Whilst technically not a metric and instead a standalone clustering technique, it can be applied to analyse clusters and sub-groups in a network.  See also “Modularity” and “Network Communities”. |
| Krackhardt's Connectedness | Global | Krackhardt’s Connectedness is one four measures used to assess the hierarchical structure of networks (Krackhardt, 1994). |
| Link Criticality | Local | Link criticality is a network performance indicator developed in transportation related research to assess the network components. In contrast to vulnerability, which identifies weak links, criticality identifies important links (Knoop et al. 2012) |
| Mixed Trophic Impact | Global | Mixed Trophic Impact (MTI) is used to quantify the relative impact a node, or component, has on other components. Used in Ecological Network Analysis |
| Modularity | Global | Modularity measures the strength of division between ‘modules’ (e.g. clusters, communities, groups) in a network (Newman, 2006). |
| Nearest Neighbour | Local | Nearest neighbour analysis compares the observed average distances between nearest neighbouring points and those of a known pattern (Lee and Wong, 2020). |
| Network Communities | Global | Networks are divided into modules or grouped in smaller clusters when the nodes of the same cluster have denser connections compared to other nodes (Tan et al. 2018).  See also “Modularity” |
| Network Flux | Global | In the context of Ecological Network Analysis, Flux analysis refers to the process of quantifying metabolic flows (Orth et al. 2010). |
| Network Motifs | Global | Motifs are recurring patterns of connections within a network that can represent broad ranges of phenomena (Milo et al. 2002). An abundance of triadic motifs in networks is significantly correlated with stability of robustness against a perturbation (Shcherrer et al. 2008). |
| Network Operation Reliability | Global | Network Operation Reliability is an index used to measure the performance of traffic in transport related networks (Jiang et al. 2016). |
| Network Spare Capacity | Global | Network spare capacity dimension is to quantify the network- wide residual capacity from planners’ perspective with an explicit consideration of travellers’ mode and route choices as well as congestion effect. These two measures can complement each other (i.e., modelling network topology and travel choice behaviours under congestion) by providing a two-dimensional characterization of transportation network redundancy from the perspective of both travellers and planners (Xu et al. 2018). It helps to answer “*how much spare capacity does the network have?”* |
| Network Stability | Global | Network stability is used to measure the efficiency of a system. The flow connection diversity is calculated using the Shannon Diversity index (Shannon and Weaver, 1949; Keil et al. 1994), reflecting the throughflows which represent total system capacity. |
| Network Utility | Global | In Ecological Network Analysis, Network Utility is a way of analysing flows within the system at high and low levels of detail in terms of the relationships and degrees of mutualism between different nodes (Liu et al. 2011). The utility method is described by Fath and Patten (1999) and Fath (2007). |
| Nodal Saturation | Local | Nodal saturation encapsulates capacity and delay and is defined as the current degree of a node divided by the maximum degree (DeLaurentis & Ayyalasomayajula, 2009). |
| Nodality | Local | Nodality is a metric, used in tandem with Centrality to measure the development strategy of a town in terms of the scale by its population size and GDP. A town with high nodality and centrality for example indicates a town with large economy and levels of development (Cui et al. 2019). |
| Node Efficiency | Local | Node efficiency describes the convenience of reaching other nodes (Zheng et al. 2020).  See also "Transition efficiency". |
| Node Strength | Local | The sum of weights attached to ties belonging to a node (Barrat et al. 2004; Opsahl et al. 2008). Useful measure for analysing weighted networks. |
| PageRank | Local | PageRank is a scalable algorithm typically used to rank web pages based on the network strucutre of links to and from web pages (Page et al. 1999). It is an adjustment of Katz Centrality. |
| Percolation Centrality | Local | Percolation centrality specifically measures the importance of nodes in terms of aiding 'percolation' in the network (Piraveenan et al. 2013). Centrality is measured as the proportion of 'percolated paths' that go through a node at a given time. A 'percolated path' is a shortest path between a pair of nodes, where the source node is percolated (i.e. the nodes state, in terms of weighting, changes. e.g. disease infection). |
| Power Centrality | Local | Power Centrality (in social networks) reflects the degree to which an individual’s status is a function of the statuses of those to whom he or she is connected to (Bonacich, 1987). |
| Random Centrality | Local | Random centrality is simply a measurement of centrality where nodes are randomly removedto assess the impact on Centrality of other nodes (Der Sarkissian et al. 2020) |
| Reciprocity | Local | Reciprocity index describes the degree to which a member has mutual ties to another member (Kilduff & Tsai, 2003; Wasserman & Faust, 1994). In direct relations between A and B, reciprocity indicator implies if A has a tie with B, it is expected of B to have a tie with A that implies ties in both directions. |
| Regionalisation | Global | The degree to which these communities are different in terms of regionalization by defining a regionalization index as the entropy in singular values of a normalized adjacency matrix (Shrestha et al. 2018) |
| Risk Criticality | Local | The risk criticality refers to the level of risk impact by looking at its magnitude and propagation impact affecting both to the community and other risks (Ongkowijoyo & Doloi, 2017). This is a composite indicator that uses in/out degree, betweenness, closeness and Eigenvector centralities. |
| Singular Value Decomposition | Local | Singular Value Decomposition (SVD) can be used to observe the interactions between nodes in a two-mode network. As a dimensionality reduction technique, it is similar to factor and correspondence analysis methods (e.g. MCA, PCA) and is useful in network analysis as it measures distances between variable (i.e. nodes) whilst measuring their contribution (W. Chen et al. 2020) |
| Status Centrality | Local | Status centrality computes the relative influence of a node within a network by measuring the number of the immediate neighbours and also all other nodes in the network that connect to the node under consideration through their immediate neighbours (Jin et al. 2014). |
| Structural Equivalence | Local | Structural equivalence means that if two nodes in a network swap their positions, the network structure will not change (Sun et al. 2019). |
| Structural Holes | Local | A structural hole is a nonredundant connection between two actors (Cui & Li, 2020). From the perspective of the overall network, it appears as a hole in the network structure (Hou & Xiao, 2015). Based on Burt's structural hole index (Burt, 1992). |
| Throughflow | Global | Throughflow is the basis of all Ecological Network Analysis(Chen et al. 2015). It is defined as the total amount of energy or matter that flows through each node (Finn, 1976). This is a fundamental feature of ecosystems and socio-economic network models (Ulanowicz, 1986). This node-level metric serves as a measure of the energy, matter, or trade volume flows in each model node, and it can be an indicator of the relative importance of each node (Borrett, 2014). The sum of the node throughflows, termed total system throughflow, is a network-level indicator of the size of the system with respect to its activity. However, the throughflow of nodes can also be examined, making this a local and global metric. |
| Transition Efficiency | Local | Transitions Efficiency represents accessibility from one node to another and is inversely proportional to the length of the shortest path between them (Zheng et al. 2020).  See also "Node efficiency". |
| Transitivity | Local | Transitivity index refers to the tendency of two members in the network to be connected if they have a common neighbourhood to complete the triple relations (Kilduff & Tsai, 2003; Wasserman & Faust, 1994). |
| Travel Alternative Diversity | Global | In the context of transportation network redundancy, travel alternative diversity dimension is to evaluate the existence of multiple modes and effective routes available for travellers, or the number of effective connections between a specific Origin-Destination (O–D) pair. Travelers might not treat all simple routes as their effective alternatives. Shorter detoured routes with an acceptable travel cost (i.e., not-too-long routes) are more likely to be considered by travellers as a reasonable substitution when the primary or secondary route is not available (Xu et al. 2018). It can answer *“how many effective redundant alternatives are there for travellers in the vent of a disruption?”.* |
| Two-step Reach | Global | Two-step reach illustrates the proportion of the total number of people in the network who can be reached by a person within one link of the people who comprise his/her immediate ties. It is considered a measure of how quickly a person can mobilise resources or convey information to others (Conte et al. 2017). |
| Unified Network Performance Measure | Global | See "Link Criticality" (Mitsakis et al. 2016). |
| z-P Parameter | Global | z-P parameter is a topological measure used to re-define hub firms instead of degree. The z-P parameter space uses a multi-criteria where one is the within-module degree z in its own community and the other one is the participation coefficient P between other communities. This measure separates nodes into seven different roles, where we focused on the “connector hubs” (see Guimera and Amaral, 2005) |

# references

Barrat A., Barthelemy M., Pastor-Satorras R., Vespignani A. (2004). The architecture of complex weighted networks. *Proceedings of the National Academy of Sciences, 101*(11), 3747-3752. [arXiv:cond-mat/0311416](http://arxiv.org/abs/cond-mat/0311416)

Bodini A. (2012). Building a systemic environmental monitoring and indicators for sustainability: What has the ecological network approach to offer? *Ecological Indicators*, *15*(1), 140–148. https://doi.org/10.1016/j.ecolind.2011.09.032

Bodini A., Bondavalli C., Allesina S. (2012). Cities as ecosystems: Growth, development and implications for sustainability. *Ecological Modelling*, *245*, 185–198. https://doi.org/10.1016/j.ecolmodel.2012.02.022

Bonacich P. (1987). Power and Centrality: A Family of Measures. *American Journal of Sociology*, *92*, 1170-1182.

Borrett S. R., Lau M. K. (2014). enaR: An R package for ecosystem network analysis. *Methods in Ecology and Evolution*, *5*(11), 1206−1213.

Burns M. C., Roca Cladera J., Moix Bergadà M. (2008). The spatial implications of the functional proximity deriving from air passenger flows between European metropolitan urban regions. *GeoJournal*, *71*(1), 37–52. https://doi.org/10.1007/s10708-008-9144-x

Burt R. S. (1995). Structural Holes: The Social Structure of Competition. Cambridge: Harvard University Press. [ISBN](https://en.wikipedia.org/wiki/ISBN_(identifier)) [978-0-674-84372-1](https://en.wikipedia.org/wiki/Special:BookSources/978-0-674-84372-1).

Calliari E., Michetti M., Farnia L., Ramieri E. (2019). A network approach for moving from planning to implementation in climate change adaptation: Evidence from southern Mexico. *Environmental Science and Policy*, *93*(November 2017), 146–157. https://doi.org/10.1016/j.envsci.2018.11.025

Carley K., Reminga J., Storrick J., Columbus D. (2011). ORA User’s Guide. Technical Report CMU-ISR-11-107, CASOS, CMU.

Cavalcanti T. V. V., Giannitsarou C. (2016). Network Cohesion. *Economic Theory*. *64*, 1- 21.

Chakraborty O., Das A., Dasgupta A., Mitra P., Ghosh S. K., Mazumder T. (2018). A multi-objective framework for analysis of road network vulnerability for relief facility location during flood hazards: A case study of relief location analysis in Bankura District, India. *Transactions in GIS*, *22*(5), 1064–1082. https://doi.org/10.1111/tgis.12314

Chen S., Chen B., Su M. (2015). Nonzero-Sum Relationships in Mitigating Urban Carbon Emissions: A Dynamic Network Simulation. *Environmental Science and Technology*, *49*(19), 11594–11603. https://doi.org/10.1021/acs.est.5b02654

Chen W., Zhang H., Comfort L. K., Tao Z. (2020). Exploring complex adaptive networks in the aftermath of the 2008 Wenchuan earthquake in China. *Safety Science*, *125*(December 2019), 104607. https://doi.org/10.1016/j.ssci.2020.104607

Chen Y., Paul G., Cohen R., Havlin S., Borgatti S. P., Liljeros F., Stanley H. E. (2007). Percolation theory and fragmentation measures in social networks. *Physica A: Statistical Mechanics and its Applications*, *378*, 11-19. doi:10.1016/j. physa.2006.11.074

Comfort L. K., Haase T. W., Ertan G., Scheinert S. R. (2020). The Dynamics of Change Following Extreme Events: Transition, Scale, and Adaptation in Systems Under Stress. *Administration and Society*, *52*(6), 827–861. https://doi.org/10.1177/0095399719869991

Conte K. P., Groen S., Loblay V., Green A., Milat A., Persson L., Innes-Hughes C., Mitchell J., Thackway S., Williams M., Hawe P. (2017). Dynamics behind the scale up of evidence-based obesity prevention: Protocol for a multi-site case study of an electronic implementation monitoring system in health promotion practice. *Implementation Science*, *12*(1), 1–10. https://doi.org/10.1186/s13012-017-0686-5

Cui J., Luo J., Kong X., Sun J., Gu J. (2019). Characterising the hierarchical structure of urban-rural system at county level using a method based on interconnection analysis. *Journal of Rural Studies*, *July*, 0–1. https://doi.org/10.1016/j.jrurstud.2019.10.013

Cui P., Li D. (2020). A SNA-based methodology for measuring the community resilience from the perspective of social capitals: Take Nanjing, China as an example. *Sustainable Cities and Society*, *53*, 101880. https://doi.org/10.1016/j.scs.2019.101880

DeLaurentis D. A., Ayyalasomayajula S. (2009). Exploring the synergy between industrial ecology and system of systems to understand complexity a case study in air transportation. *Journal of Industrial Ecology*, *13*(2), 247–263. https://doi.org/10.1111/j.1530-9290.2009.00121.x

Der Sarkissian R., Abdallah C., Zaninetti J. M., Najem S. (2020). Modelling intra-dependencies to assess road network resilience to natural hazards. *Natural Hazards*, *103*(1), 121–137. https://doi.org/10.1007/s11069-020-03962-5

Derudder B., Taylor P. (2005). The cliquishness of world cities. *Global Networks*, *5*(1), 71–91. https://doi.org/10.1111/j.1471-0374.2005.00108.x

Diestel R. (2005).[Graph Theory](http://diestel-graph-theory.com/GrTh.html). 5^th^ edn, Graduate Texts in Mathematics. p. 12.

Everett M., Borgatti S. P. (2005). Ego network betweenness. *Social Networks*, *27*(1), 31–38. https://doi.org/10.1016/j.socnet.2004.11.007

Fath B. D., Patten B. C. (1999). Review of the foundations of network environ analysis. *Ecosystems.* *2*, 167–179

Fath B. D. (2007). Community-level relations and network mutualism. *Ecological Modelling*. , 56–67.

Finn J. T., (1976). Measures of ecosystem structure and function derived from analysis of flows. *Journal of Theoretical Biology*. *56*, 363–380

Freeman L. C. 1977. A Set of Measures of Centrality Based on Betweenness. *Sociometry* *40*(1), 35-41

Freeman L., C. (1978). Centrality in Social Networks: Conceptual Clarification. *Social Networks*. *1*(3), 215 – 239.

Frey B. B. (2016). Network Density. The SAGE Encyclopaedia of Educational Research, Measurement and Evaluation. SAGE. <https://dx.doi.org/10.4135/9781506326139.n470>

Gao P., Hung J., Xu Y. (2020). A k-core decomposition-based opinion leaders identifying method and clustering-based consensus model for large-scale group decision making. *Computers and Industrial Engineering*, *150*(September), 106842. https://doi.org/10.1016/j.cie.2020.106842

Ghanbari R, Jalili M., Yu X. (2018). Correlation of cascade failures and centrality measures in complex net-works. *Future Generation Computer Systems* *83*, 390–400. <https://doi.org/10.1016/j.future.2017.09.007>

Golbeck J. (2013). Network Structure and Measures. *Analyzing the Social Web*, *5*, 25–44. https://doi.org/10.1016/b978-0-12-405531-5.00003-1

Golbeck J. (2015). Analyzing networks. *Introduction to Social Media Investigation*, 221–235. https://doi.org/10.1016/b978-0-12-801656-5.00021-4

Guimera R., Amaral L. A. N. (2005). Functional cartography of complex metabolic networks. *Nature*, *433*, 895–900

Han R., Cao H., Liu Z. (2018). Studying the urban hierarchical pattern and spatial structure of China using a synthesized gravity model. *Science China Earth Sciences*, *61*(12), 1818–1831. https://doi.org/10.1007/s11430-016-9191-5

Hanson W. R., Ford R. (2010). Complexity leadership in healthcare: Leader network awareness. *Procedia - Social and Behavioral Sciences*, *2*(4), 6587–6596. https://doi.org/10.1016/j.sbspro.2010.04.069

He D., Sun Z., Gao P. (2019). Development of economic integration in the central Yangtze River Megaregion from the perspective of urban network evolution. *Sustainability (Switzerland)*, *11*(19). https://doi.org/10.3390/su11195401

Hou J., Xiao R. (2015). Identifying critical success factors of linkage mechanism between government and non-profit in the geo-disaster emergency decision. *International Journal of Emergency Management*, *11*(2): 146-168

Hu X., Wang C., Wu J., & Stanley H. E. (2020). Understanding interurban networks from a multiplexity perspective. *Cities*, *99*(July 2019), 102625. https://doi.org/10.1016/j.cities.2020.102625

Jiang R., Chen J. Y., Ding Z. J., Ao D. C., Hu M., Bin Gao, Z. Y., Jia B. (2016). Network operation reliability in a Manhattan-like urban system with adaptive traffic lights. *Transportation Research Part C: Emerging Technologies*, *69*, 527–547. https://doi.org/10.1016/j.trc.2016.01.006

Jin L., Jiong W., Yang D., Huaping W., Wei D. (2014). A simulation study for emergency/disaster management by applying complex networks theory. *Journal of Applied Research and Technology*, *12*(2), 223–229. https://doi.org/10.1016/S1665-6423(14)72338-7

Keil R. G., Montluçon D. B., Prahl, F .G., Hedges J. I. (1994). Sorptive preservation of labile or- ganic matter in marine sediments. *Nature*. *370*(6490), 549

Kilduff M., Tsai W. (2003). Social network and organization. London: SAGE

Knoop V. L., Snelder M., van Zuylen H. J., Hoogendoorn S.P (2012) Link-level vulnerability indicators for real-world networks. *Transportation Research. Part A Policy and Practice*. *46*(5), 843–854.

Krackhardt D., Stern R. (1988). Informal networks and organisational crises: An experimental simulation. *Social Psychology Quarterly*, *51*(2), 123–140

Krackhardt D. (1994). Graph Theoretical Dimensions of Informal Organizations. Lawrence Erlbaum, Hillsdale, NJ, 89–111

Laine C., R. (1995). The Herfindahl-Hirschman index: a concentration measure taking the consumer's point of view. The antitrust bulletin. *423*. <https://doi.org/10.1177%2F0003603X9504000206>

Latora V., Marchiori M. (2003). Economic small-world behavior in weighted networks. *European Physical Journal B*, *32*(2), 249–263. https://doi.org/10.1140/epjb/e2003-00095-5

Lee J., Wong D. (2000). Statistical analysis with ArcView GIS. New York: Wiley

Liu G. Y., Yang Z. F., Chen B., Zhang Y. (2011). Ecological network determination of sectoral linkages, utility relations and structural characteristics on urban ecological economic system. *Ecological Modelling*, *222*(15), 2825–2834. https://doi.org/10.1016/j.ecolmodel.2011.04.034

Makagon M., M., McCowan B., Mench J., A. (2012). How can social network analysis contribute to social behaviour research in applied ethology? *Applied Animal Behavhavioural Science. 138*(3 - 4). doi: [10.1016/j.applanim.2012.02.003](https://dx.doi.org/10.1016%2Fj.applanim.2012.02.003)

March J. G. (1991). Exploration and exploitation in organizational learning. *Organisational Science.* *2*(1), 71–87

Milo R, Shen-Orr S, Itzkovitz S, Kashtan N, Chklovskii D, Alon U (2002) Network motifs: simple building blocks of complex networks. *Science*, *298*(5594), 824–827.

Murray-Tuite P. M., Mahmassani H. S. (2004). Methodology for determining vulnerable links in a transportation network. *Transportation Research Record*. *1882*, Transportation Research Board, Washington, D.C., 88–96.

Murray-tuite P. (2007). *Unplanned Disruptions*. *133*(1), 9–17.

Modrak V., Marton D. (2013). Development of Metrics and a Complexity Scale for the Topology of Assembly Supply Chains. *Entropy*, *15*, 4285–4299.

Neal Z. (2011). Differentiating centrality and power in the world city network. *Urban Studies*, *48*(13), 2733–2748. doi: 10.1177/0042098010388954

Newman M. (2003). The structure and function of complex networks. *SIAM Review*. *45*(2):167–256. https: //doi.org/10.1137/ S003614450342480

Newman M. E. (2002). Assortative mixing in networks. *Physical Review Letters,* *89* (20): 208701. <https://doi.org/10.1103/PhysRevLett.89.208701>

Newman M. E. J. (2006). Modularity and community structure in networks. *Proceedings of the National Academy of Sciences of the United States of America*. *103*(23), 8577-8582. <https://doi.org/10.1073/pnas.0601602103>

Odum E. P. (1969). The strategy of ecosystem development. *Science.* *164*, 262–269

Opsahl T., Agneessens F., Skvoretz J. (2010). [Node centrality in weighted networks: Generalizing degree and shortest paths](https://toreopsahl.com/2010/04/21/article-node-centrality-in-weighted-networks-generalizing-degree-and-shortest-paths/). *Social Networks*. *32*, 245-251.

Orth J. D., Thiele I., Palsson B. Ø. (2010). What is flux balance analysis?. *Nature biotechnology*, *28*(3), 245–248. https://doi.org/10.1038/nbt.1614

Ongkowijoyo C., Doloi H. (2017). Determining critical infrastructure risks using social network analysis. *International Journal of Disaster Resilience in the Built Environment*, *8*(1), 5–26. https://doi.org/10.1108/IJDRBE-05-2016-0016

Page L, Brin S, Motwani R, Winograd T (1999) The PageRank citation ranking: bringing order to the web. Technical Report, Stanford InfoLab.

Piraveenan M, Prokopenko M, Hossain L (2013) Percolation Centrality: Quantifying Graph-Theoretic Impact of Nodes during Percolation in Networks. PLoS ONE 8(1): e53095. <https://doi.org/10.1371/journal.pone.0053095>

Reminga J., Carley K. (2003). Measures in ORA. CASOS, Carnegie Mellon University.

Schramski J. R., Gattie D. K., Patten B. C., Borrett S. R., Fath B. D., Thomas C. R., Whipple S. J. (2006). Indirect effects and distributed control in ecosystems: distributed control in the environ networks of a seven-compartment model of nitrogen flow in the Neuse River Estuary, USA—steady-state analysis. *Ecological Modelling*. *194*, 189–201. <https://doi.org/10.1016/J.ECOLMODEL.2005.10.012>.

Schreiber C., Carley K. (2008). Network leadership: Leading for learning and adaptability. In M. Uhl-Bien & R. Marion (Eds.), Complexity Leadership, Part I: Conceptual Foundations, Information Age Publishing. 291–332

Scherrer A., Borgnat P., Fleury E., Guillaume J. L., Robardet C. (2008) Description and simulation of dynamic mobility networks. *Computer Netw orks*. *52*(15), 2842–2858

Shannon C. E., Weaver W. (1949). The Mathematical Theory of Communication. University of Illinois Press, Urbana

Shrestha M., Scarpino S. V., Edwards E. M., Greenberg L. T., Horbar J. D. (2018). The interhospital transfer network for very low birth weight infants in the United States. *EPJ Data Science*, *7*(1). https://doi.org/10.1140/epjds/s13688-018-0155-7

Scott J. (1991) Social network analysis, London: Sage. 171.

Seidman S. B. (1983). Network structure and minimum degree. *Social Networks*, *5,* 269–287.

Sorenson O. (2003). Interdependence and adaptability: organizational learning and the long-term effect of integration. *Managerial Science.* *49*(4), 446–463

Strano E., Shai S., Dobson S., Barthelemy M. (2015). Multiplex networks in metropolitan areas: Generic features and local effects. *Journal of the Royal Society Interface*, 12, 20150651.

Stewart J. Q. (1948). Demographic gravitation: Evidence and applications. *Sociometry*, 11-31

Sun Q., Wang S., Zhang K., Ma F., Guo X., Li T. (2019). Spatial pattern of urban system based on gravity model and whole network analysis in eight urban agglomerations of China. *Mathematical Problems in Engineering*, *2019*. https://doi.org/10.1155/2019/6509726

Tan L. M., Arbabi H., Li Q., Sheng Y., Densley Tingley, D., Mayfield M., Coca D. (2018). Ecological network analysis on intra-city metabolism of functional urban areas in England and Wales. *Resources, Conservation and Recycling*, *138*(May), 172–182. https://doi.org/10.1016/j.resconrec.2018.06.010

Tang P., Lai S. (2019). A framework for managing public security risks with complex interactions in cities and its application evidenced from Shenzhen City in China. *Cities*, *95*(April), 102390. https://doi.org/10.1016/j.cities.2019.102390

Tribus M., McIrvine E. C. (1971). Energy and information. *Scientific American.* *225*, 179–188

Ulanowicz R. E. (1980). An hypothesis on the development of natural communities. *Journal of Theoretical Biology.* *85*, 223–245.

Ulanowicz, R. E. (1986). Growth and Development. Ecosystem Phenomenology. Springer, New York.

Ulanowicz R. E., Puccia C. J. (1990). Mixed Trophic Impacts in Ecosystems. *JSTOR*. *5*(1) 7 – 16. <https://www.jstor.org/stable/43461017>

Von Gnechten R., Wang J., Konar M., Baylis K., Anderson P., Giroux S., Jackson N. D., Evans T. (2020). A gravity model and network analysis of household food sharing in Zambia. *Environmental Research Letters*, *15*(11). https://doi.org/10.1088/1748-9326/abbe44

Vragovic I., Louis E. Diaz-Guilera A. (2005). Efficiency of informational transfer in regular and complex networks, *Physical Review E*, *71*(3), 036122, https://doi.org/10.1103/PhysRevE.71.036122

Watts, D. J., Strogatz, S. (1998). Collective dynamics of 'small-world' networks. [*Nature*](https://en.wikipedia.org/wiki/Nature_(journal)). *393*(6684): 440–442 [doi](https://en.wikipedia.org/wiki/Doi_(identifier)):[10.1038/30918](https://doi.org/10.1038%2F30918)

Wang T., Yue W., Ye X., Liu Y., Lu D. (2020). Re-evaluating polycentric urban structure: A functional linkage perspective. *Cities*, *101*(January), 102672. https://doi.org/10.1016/j.cities.2020.102672

Wasserman S., Faust K. (1994). Social network analysis: Methods and applications. 23–188, Cambridge. Cambridge University Press

Xu X., Chen A., Jansuwan S., Yang C., & Ryu S. (2018). Transportation network redundancy: Complementary measures and computational methods. *Transportation Research Part B: Methodological*, *114*, 68–85. https://doi.org/10.1016/j.trb.2018.05.014

Zheng W., Kuang A., Wang X., Chen J. (2020). Measuring Network Configuration of the Yangtze River Middle Reaches Urban Agglomeration: Based on Modified Radiation Model. *Chinese Geographical Science*, *30*(4), 677–694. https://doi.org/10.1007/s11769-020-1131-2

Zuo Y., Kajikawa Y. (2017). Toward a theory of industrial supply networks: A multi-level perspective via network analysis. *Entropy*, *19*(8), 1–23. https://doi.org/10.3390/e19080382
